# Exploring broilers and native fowls of Andaman and Nicobar Islands as a source of β-lactamase-producing *Enterobacteriaceae* even with limited anthropogenic activities and docking-based identification of catalytic domains in novel β-lactamase variants

**DOI:** 10.3389/fvets.2022.1075133

**Published:** 2023-01-05

**Authors:** Sneha Bhowmick, Surajit Pal, Jai Sunder, T. Sujatha, Arun Kumar De, Tousif Mondal, Abhishek D. Singh, Siddhartha Narayan Joardar, Kunal Batabyal, Tapan Kumar Dutta, Samiran Bandyopadhyay, Ananda Tiwari, Indranil Samanta

**Affiliations:** ^1^Department of Veterinary Microbiology, West Bengal University of Animal and Fishery Sciences, Kolkata, West Bengal, India; ^2^Animal Science Division, ICAR-Central Island Agricultural Research Institute, Port Blair, Andaman and Nicobar Islands, India; ^3^Department of Veterinary Public Health, West Bengal University of Animal and Fishery Sciences, Kolkata, West Bengal, India; ^4^Department of Veterinary Microbiology, Central Agricultural University, Aizawl, Mizoram, India; ^5^ICAR-Indian Veterinary Research Institute, Eastern Regional Station, Kolkata, West Bengal, India; ^6^Department of Food Hygiene and Environmental Health, University of Helsinki, Helsinki, Finland

**Keywords:** Andaman and Nicobar, docking, clonal, ESBL, poultry

## Abstract

**Objectives:**

The present study was conducted to detect the occurrence of β-lactamase and biofilm-producing *Escherichia coli, Salmonella*, and *Klebsiella* in broilers and native fowl reared in the Andaman and Nicobar Islands, India. The study also included molecular docking experiments to confirm the nature of the catalytic domains found in the β-lactamase variants obtained and to reveal the clonal relationship of the isolates with human clinical strains from the database.

**Materials and methods:**

A total of 199 cloacal swabs were collected from five poultry breeds/varieties (broiler, *Vanraja, Desi, Nicobari*, and layer) in three districts of the Andaman and Nicobar Islands. *E. coli, Salmonella enterica*, and *Klebsiella pneumoniae* were isolated by standard techniques and confirmed by PCR. Phenotypical β-lactamase producers were identified by a double-disc test. The genes (*bla*_CTX_, *bla*_SHV_, *bla*_*TEM*_, and *bla*_AmpC_) were screened, and selected sequences of β-lactamase variants were submitted to DDBJ. Homology modeling, model validation, and active site identification of different β-lactamase variants were done by the SWISS-MODEL. Molecular docking was performed to identify the catalytic domains of the β-lactamase variants. The selected β-lactamase sequences were compared with the Indian ESBL sequences from human clinical strains in NCBI-GenBank.

**Results:**

In total, 425 *Enterobacteriaceae* strains were isolated from the collected samples. *Klebsiella pneumoniae* (42.58%) was found to be the most prevalent, followed by *Salmonella enterica* (30.82%) and *E. coli* (26.58%). The phenotypical antibiogram of all 425 isolates showed the highest resistance against oxytetracycline (61–76%) and the lowest against gentamicin (15–20%). Phenotypical production of β-lactamase enzymes was observed in 141 (33.38%) isolates. The isolation rate of β-lactamase producing *E. coli, Salmonella enterica*, and *Klebsiella pneumoniae* was significantly higher (*p* < 0.05) in the birds reared in the South Andaman district (25.6, 17.5, and 18.7%, respectively) than in Nicobar (11.5, 7.6, 7.1%, respectively). Genotyping of the β-lactamase-producing isolates revealed the maximum possession of *bla*_TEM_, followed by *bla*_SHV_ and *bla*_CTX − M_. The nucleotide sequences were found to be similar with *bla*_CTX − M−15_, *bla*_SHV − 11_, *bla*_SHV − 27_, *bla*_SHV − 228_, *bla*_TEM − 1_, and *bla*_AmpC_ in BLAST search. Distribution of studied biofilm-associated genes in *Enterobacteriaceae* strains from different varieties of the birds revealed that the layer birds had the maximum possession, followed by *Vanraja, Desi*, broilers, and *Nicobari* fowls. The phylogenetic analysis of selected sequences revealed a partial clonal relationship with human clinical strains of the Indian subcontinent. Molecular docking depicted the Gibbs free energy release for 10 different macromolecules (proteins) and ligand (antibiotic) complexes, ranging from −8.1 (SHV-27 + cefotaxime) to −7 (TEM-1 + cefotaxime) kcal/mol.

**Conclusion and relevance:**

The study revealed β-lactamase variants circulating in the fowl population of the Andaman and Nicobar Islands (India), even in remote places with low anthropogenic activity. Most of the strains possessed *bla*_TEM − 1_, followed by *bla*_CTX − M−15_. Possession of *bla*_SHV − 11_, *bla*_SHV − 27_, and *bla*_SHV − 228_ in poultry *Enterobacteriaceae* strains was not reported earlier from any part of the world. The phylogenetic analysis revealed a partial clonal relationship of β-lactamase sequences with the human clinical strains isolated from the Indian subcontinent.

## Introduction

Antimicrobial resistance in livestock is a global challenge as the bacteria possessing the resistance genes can be disseminated into the human food chain through cross-contamination by means of occupational exposure, contaminated environment, and consumption of animal-origin foods. Extended spectrum-β-lactamase (ESBL) and AmpC-β-lactamase (ACBL) producing *Enterobacteriaceae* are the most reported antimicrobial-resistant bacteria in humans and livestock in the last two decades (1). Poultry was identified as the major reservoir of ESBL-producing *Enterobacteriaceae* in comparison to pigs, cattle, and other members of the livestock family (2). The poultry as a reservoir of ESBL-producing bacteria acts as a challenge for the farmers and slaughterhouse workers or meat vendors, as increased gut colonization of the resistant bacteria was detected in people who had more contact with the birds than the community (3). A recent whole-genome sequencing-based study also evidenced the transmission of ESBL-producing bacteria from poultry to the human population (4).

The ESBL enzyme generates resistance to β-lactam antibiotics, including higher-generation cephalosporins and monobactams. AmpC β-lactamase-producing bacteria (ACBL) can develop resistance against β-lactam antibiotics in addition to β-lactamase inhibitors like clavulanic acid. There are three classical ESBLs, i.e., TEM (except TEM-1, TEM-2, and TEM-13), SHV (except SHV-1, SHV-2, and SHV-11), and CTX-M (5). CTX-M-15 is the most common ESBL genotype prevalent currently among the human clinical isolates with a rising trend of CTX-M-1, frequently originating from livestock and poultry (6). Poultry acts as the major reservoir of CTX-M-1, SHV-12, TEM-52, and AmpC β-lactamases (7).

Anthropogenic activities were found to be associated with the development of an ESBL-“resistome” in the environment including aquatic settings either due to the dissemination of ESBL-determinants or the bacteria carrying the genes associated with direct human activities and/or the release of the antimicrobials in the sub-therapeutic level in the environment because of indirect human activities (8–10). Persistence of ESBL-producers on the abiotic or biotic surface, associated with the development of “resistome”, is dependent on the capacity to form biofilms, as they help in the survival of the bacterial colony against physical and chemical stresses, including disinfectants, host phagocytosis, and antibiotics (11). However, a recent study identified antimicrobial resistance genes in the commensals present in soil exposed to low anthropogenic activities (12).

Several studies found variants of ESBL in *Enterobacteriaceae* in healthy or diseased poultry birds (6, 13), but limited literature is available about the affinity of the β-lactamases for the precise class of cephalosporins. The present study was conducted to detect the presence of β-lactamase and biofilm-producing *Escherichia coli, Salmonella*, and *Klebsiella* in broilers and backyard or native fowl reared in the Andaman and Nicobar Islands (India), even in remote places with low anthropogenic activities. The study also included molecular docking experiments to confirm the nature of the catalytic domains in β-lactamase variants (*bla*_CTX − M_, *bla*_SHV_, and *bla*_TEM_) and phylogenetic analysis to reveal the clonal relationship of the poultry-origin *Enterobacteriaceae* isolates with human clinical strains from the GenBank database. *Enterobacteriaceae* was selected as the study bacteria as the family is included in the World Health Organization (WHO) global priority list under “critical” category as an indicator of antibiotic resistance.

## Materials and methods

### Sampling

During the period from November 2019 to January 2021, a total of 199 cloacal swabs (Table 1) were collected from five poultry breeds or varieties (broiler, *Vanraja, Desi, Nicobari*, and layer) irrespective of age and sex in three different districts of the Andaman and Nicobar Islands (India), i.e., South Andaman (S/A) (11.74°N/92.65°E), North and Middle Andaman (N & M/A) (12.65°N/92.89°E), and Nicobar (C/N) (7.12°N/93.78°E). The sample size varied between the districts depending on the accessibility and willingness of the farmers to join the study. The collected swabs taken from live birds were properly labeled and were aseptically transported, maintaining the cold chain, into the bacteriology laboratory of the Animal Science Division, ICAR-CIARI, Andaman and Nicobar Islands (India).

**Table 1:** Distribution of ESBL-producing *E. coli, Salmonella*, and *Klebsiella* in three districts of Andaman and Nicobar Islands (India).

**District**	**Breed**	**Number of collected samples**	**Number of *E.coli* isolates**	**Number of ESBL-*E.coli* isolates (%)**	**Number of *Salmonella* isolates (%)**	**Number of ESBL-*Salmonella* isolates (%)**	**Number of *Klebsiella* isolates (%)**	**Number of ESBL-*Klebsiella* isolates (%)**
South Andaman	*Vanraja*	18	14	8	14	3	17	8
	*Desi*	20	14	10	14	2	20	4
	*Nicobari*	20	14	6	11	5	19	2
	Layer	12	9	3	6	2	12	3
	Broiler	30	6	2	27	11	29	17
	Sub-Total	100	57 (57/113, 50.44%)	29^*^ (29/113, 25.6%)	72 (72/131, 54.96%)	23^a^ (23/131, 17.55%)	97 (97/181, 53.59%)	34^b^ (34/181, 18.78%)
N&M Andaman	*Vanraja*	14	7	3	7	2	12	5
	*Desi*	35	7	4	13	1	23	4
	Sub-Total	49	14 (14/113, 12.38%)	7 (7/113, 6.19%)	20 (20/131, 15.26%)	3 (3/131, 2.29%)	35 (35/181, 19.33%)	9 (9/181, 4.97%)
Nicobar	*Nicobari*	50	42 (42/113, 37.16%)	13^*^ (13/113, 11.5%)	39 (39/131, 29.77%)	10^a^ (10/131, 7.63%)	49 (49/181, 27.07%)	13^b^ (13/181, 7.18%)
	Total	199	113 (113/425, 26.58%)	49 (49/113, 43.36%)	131 (131/425, 30.82%)	36 (36/131, 27.48%)	181 (181/425, 42.58%)	56 (56/181, 30.93%)

* Differs significantly at a p-value of <0.05;

a Differs significantly at a p-value of <0.05;

b Differs significantly at a p-value of <0.05.

No clinical symptoms were reported by the farmers during the collection of cloacal swabs from the birds. The contract farmers reared the broilers in medium-sized flocks (100–200 birds) with the guidelines, feed, vaccines, and medicines (including antibiotics like doxycycline, neomycin, and cephalexin) provided by the enterprise. The backyard farmers reared *Vanraja*, layer birds, and native fowls such as *Desi* and *Nicobari* in small flocks consisting of 15–20 birds per household with occasional exposure to tetracyclines, neomycin, and fluoroquinolones for therapy under the guidance of local veterinarians, para-veterinarians, and drug shop owners. The backyard farmers reared the birds under a semi-intensive system with daytime roaming around the houses and overnight shelter at the farmer's house. No commercial feed mixture was detected to have been used for feeding. The contract farmers prepared a separate bamboo or brick poultry shed and used feeders and waterers, with occasional cleaning and disinfection of the shed with formalin.

### Isolation, identification, and PCR-based confirmation of *Escherichia coli, Salmonella*, and *Klebsiella*

The swab samples were transported in a sterile transport medium (transport liquid medium, HiMedia, India) and inoculated into the nutrient broth (HiMedia, India) and incubated at 37°C for 24 h. The loopful of overnight growth was streaked onto MacConkey agar (HiMedia, India) and incubated at 37°C for 24 h. The selected single pink colonies were transferred into eosin methylene blue (EMB) agar (HiMedia, India) and incubated at 37°C for 24 h. The single colonies with a greenish metallic sheen were selected for further morphological and biochemical identification (14). For the isolation of *Salmonella*, the swab samples collected were enriched with overnight growth in selenite broth (HiMedia, India) at 37°C. The loopful of growth was streaked onto brilliant green agar (BGA) (HiMedia, India) and incubated at 37°C. The single reddish colonies were selected for further morphological and biochemical identification (14). Similarly, for the isolation of *Klebsiella*, the growth in nutrient broth was streaked into *Klebsiella* selective agar (HiMedia, India) and incubated at 37°C. The single purple magenta colonies were considered for further morphological and biochemical identification (14). The tentatively identified *E. coli*, S*almonella*, and *Klebsiella* isolates were confirmed by 16SrRNA gene-specific PCR (15, 16). *Klebsiella pneumoniae* was also identified by specific PCR with the *Klebsiella* species isolates (17). The PCR products were agarose gel electrophoresed containing ethidium bromide, and the gel was visualized and documented in a gel documentation system (Labmate Asia, India).

### Antibiogram

All the *E. coli, Salmonella enterica*, and *Klebsiella pneumoniae* isolates were screened for antibiotic sensitivity with ceftazidime (CAZ) (30 μg), cefotaxime (CTX) (30 μg), ceftriaxone (CTR) (30 μg), cefpodoxime (CPD) (10 μg), cefoxitin (CX) (30 μg), aztreonam (AT) (30 μg), erythromycin (E) (15 μg), tetracycline (TE) (30 μg), chloramphenicol (C) (30 μg), amoxicillin/clavulanic acid (AMC) (20/10 μg), gentamicin (GEN) (10 μg), sulphafurazole (SF) (300 μg), ampicillin/cloxacillin (AX) (10 μg), ampicillin (AMP) (10 μg), co-trimoxazole (COT) (23.75/1.25 μg), amikacin (AK) (30 μg), ciprofloxacin (CIP) (5 μg), and oxytetracycline (O) (30 μg). The interpretation of the susceptibility or resistance was calculated as per the CLSI recommendation (18).

### Double disc diffusion test

The bacterial isolates with a zone of inhibition (ZOI) diameter of ≤ 22 mm for ceftazidime, ≤ 27 mm for cefotaxime, ≤ 25 mm for ceftriaxone, ≤ 17 mm for cefpodoxime, and ≤ 27 mm for aztreonam were considered for disc diffusion testing to detect phenotypical ESBL or AmpC production. For confirmation of ESBL production, the isolates that showed an increase of ≥5 mm in ZOI diameter when tested with CTZ and CAZ alone and in combination with ceftazidime/clavulanic acid (CAC/CAZ) (30/10 μg) and cefotaxime/clavulanic acid (CEC/CTX) (30 /10 μg) (18).

Cefoxitin (CX) (30 μg) disc screening was used for the initial detection of AmpC producers, and the isolates with ZOI diameter ≥18 mm were considered for the cefoxitin-cloxacillin double disc test. For confirmation of AmpC production, the isolates showed an increase of ≥4 mm in ZOI diameter when tested with cefoxitin alone and in combination with cefoxitin/cloxacillin (19).

### PCR-based detection of ESBL and chromosomal AmpC genes

All the isolates showing phenotypical β-lactamase production were screened for the presence of *bla*_CTX − M_, *bla*_SHV_, *bla*_*TEM*_, and *bla*_AmpC_ genes by PCR (20, 21). The PCR products were electrophoresed with ethidium bromide (0.5 μg/ml) and the gel was visualized and documented in a gel documentation system (Labmate Asia, India). The commercial source (Xcelris Genomics, India) was used for the sequencing of selected PCR products as representative of each breed or variety of the birds or the districts studied. The sequence homology was detected by the standard nucleotide BLAST algorithm (https://blast.ncbi.nlm.nih.gov/Blast.cgi?CMD=Web&PAGE_TYPE=BlastHome). The sequences were submitted to the DNA Data Bank of Japan (DDBJ; www.ddbj.nig.ac.jp).

### Detection of biofilm-associated genes

All the 425 isolates were subjected to PCR-based detection of biofilm-associated genes, namely, *csgA, sdiA*, and *rpoS* (22, 23). The commercial source (Xcelris Genomics, India) was used for the sequencing of selected PCR products. The sequence homology was detected by the standard nucleotide BLAST algorithm (https://blast.ncbi.nlm.nih.gov/Blast.cgi?CMD=Web&PAGE_TYPE=BlastHome).

### Homology modeling, model validation, and active site identification of different ESBL variants

Available PDB structures of CTX-M-15 (PDB id: 4HBU), SHV-11 (PDB id: 6NFD), and TEM-1 (PDB id: 1BTL) were pulled out from the Research Collaboratory for Structural Bioinformatics Protein Data Bank (RCSB-PDB) database (https://www.rcsb.org/). A position-specific iterated basic local alignment search tool (PSI-BLAST) was performed to find out suitable templates for SHV-28 (template PDB id: 3D4F) and SHV-228 (template PDB id: 3OPL) (https://www.ebi.ac.uk/Tools/sss/psiblast/). Protein homology modeling was performed by using the SWISS-MODEL server (https://swissmodel.expasy.org/). Other structural assessments and stereochemical qualities (Supplementary Figure 1) were verified by the PROCHECK server (https://www.ebi.ac.uk/thornton-srv/software/PROCHECK/). Catalytic active sites for the crystal structures and the modeled proteins were further deposited to DoGSiteScorer, a web server for automatic binding site detection, under the proteins plus (http://dogsite.zbh; uni-hamburg.de/) to get the potential pockets for molecular docking analyses (Supplementary Figure 2).

### Docking of third-generation cephalosporins with the ESBL variants

Molecular docking was performed on the Autodock Vina Windows Desktop Suite (https://autodock.scripps.edu/download-autodock4/) as described earlier (24). Three-dimensional SDF file structures of cefotaxime (C_16_H_17_N_5_O_7_S_2_; PubChem id: 5742673) and cefpodoxime (C_15_H_17_N_5_O_6_S_2_; PubChem id: 6335986) were retrieved from the PubChem database (https://pubchem.ncbi.nlm.nih.gov/). Receptor energy minimization was done in Swiss-PdbViewer (https://spdbv.unil.ch/energy_tut.html), and the ligand structures were optimized by the Avogadro desktop suite (https://avogadro.cc/). Two-dimensional macromolecule + ligand complexes were visualized by LIGPLOT (https://www.ebi.ac.uk/thornton-srv/software/LigPlus/install.html) analysis, and 3D complexes were made in the PyMOL (https://www.schrodinger.com/products/pymol) desktop suite.

### Partial clonal relationship of poultry origin β-lactamases producing *Enterobacteriaceae* strains with human clinical isolates

The selected β-lactamase sequences from the present study were compared with the ESBL sequences of clinical *Enterobacteriaceae* strains isolated from human patients in India and Indian subcontinents (Bangladesh, Myanmar, China, Thailand), available in the NCBI-Genbank database (National Centre for Biotechnology Information; https://www.ncbi.nlm.nih.gov/genbank/). The phylogenetic tree was constructed by the maximum likelihood (ML) method using molecular evolutionary genetics analysis (MEGA-X; https://www.megasoftware.net/) and analyzed in iTOL v6 (https://itol.embl.de/).

### Statistical analysis

The chi-square test (SPSS Inc.) was applied to reveal the statistical differences in the occurrence of β-lactamase-producing *E. coli, Salmonella*, and *Klebsiella* strains among the studied fowl population reared in the South Andaman and Nicobar districts.

## Results

In total, 425 *Enterobacteriaceae* strains were isolated from the collected samples (*n* = 199). *K. pneumoniae* (42.58%) was found to be the most prevalent, followed by *Salmonella enterica* (30.82%) and *E. coli* (26.58%) (Table 1). *E. coli, Salmonella*, and *Klebsiella* were tentatively identified by biochemical tests and confirmed with 16S-rRNA gene-specific PCR.

**Figure 1:**
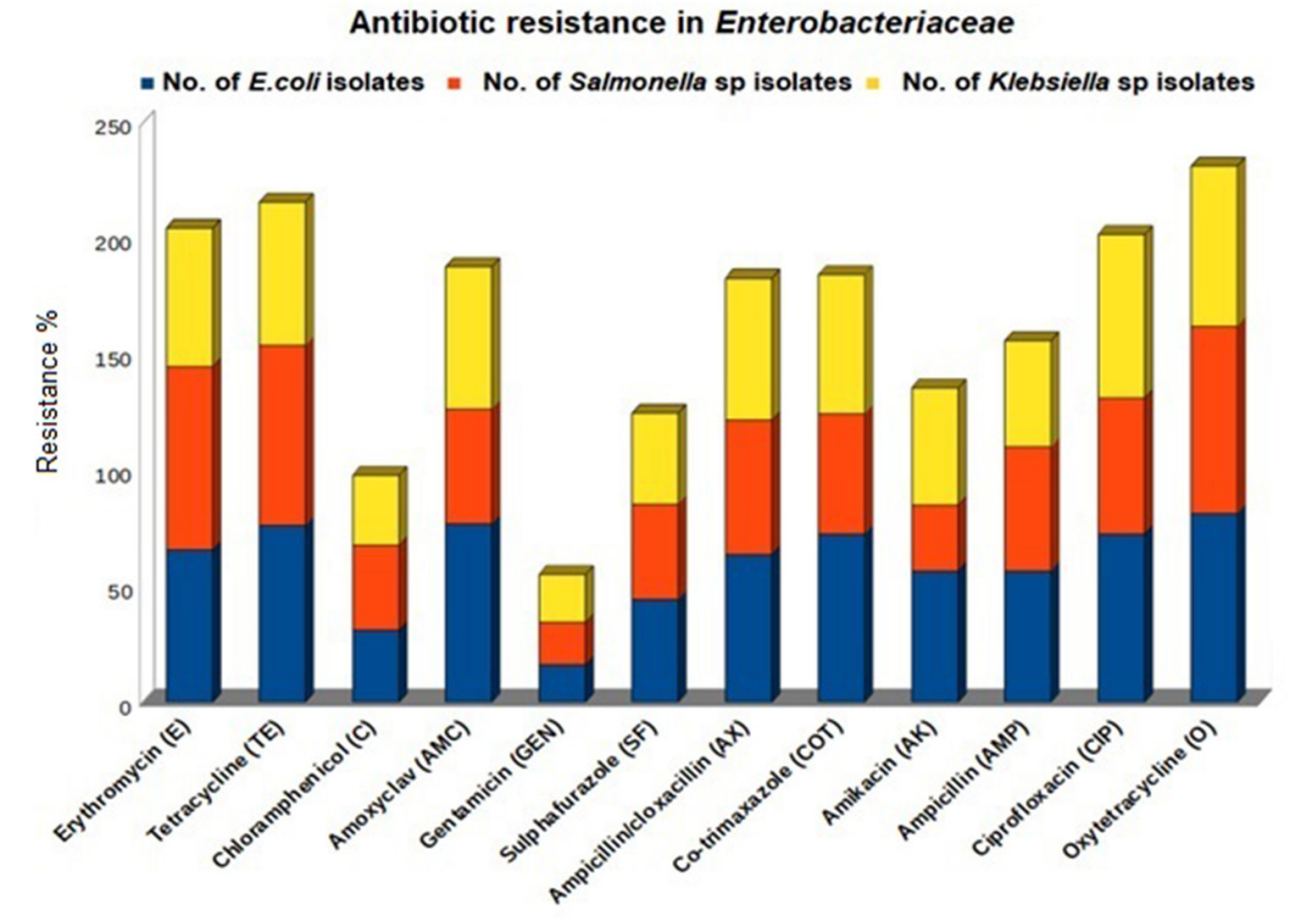
Phenotypical antibiotic resistance profile of bacterial strains isolated from different birds reared in Andaman and Nicobar Islands (India).

Phenotypical antibiotic resistance profiling of all 425 isolates showed the highest resistance against oxytetracycline (61–76%), amoxicillin/clavulanic acid (61–76%), and co-trimoxazole (60–72%), and the lowest resistance was observed against gentamicin (15–20%). *E. coli* (81.42%) and *Salmonella* (80.92%) showed the highest phenotypical resistance against oxytetracycline, whereas *Klebsiella* showed the highest resistance against ciprofloxacin (70.72%) (Table 2; Figure 1).

**Table 2:** Phenotypical antibiotic resistance in *Enterobacteriaceae* strains isolated from poultry in Andaman and Nicobar Islands (India).

**Antibiotics**	***E. coli* (%) (*n =* 113)**	***S. enterica* (%) (*n =* 131)**	***K. pneumoniae* (%) (*n =* 181)**
Erythromycin (E)	74 (65.49%)	104 (79.395%)	108 (59.67%)
Tetracycline (TE)	86 (76.11%)	102 (77.86%)	112 (61.88%)
Chloramphenicol (C)	35 (30.97%)	48 (36.64%)	55 (30.39%)
Amoxicillin/clavulanic acid (AMC)	87 (76.99%)	65 (49.62%)	111 (61.33%)
Gentamicin (GEN)	18 (15.93%)	24 (18.32%)	38 (20.99%)
Sulphafurazole (SF)	50 (44.25%)	54 (41.22%)	71 (39.23%)
Ampicillin/cloxacillin (AX)	72 (63.72%)	76 (58.02%)	111 (61.33%)
Co-trimoxazole (COT)	82 (72.57%)	68 (51.91%)	109 (60.22%)
Amikacin (AK)	43 (56.64%)	37 (28.24%)	92 (50.83%)
Ampicillin (AMP)	64 (56.64%)	70 (53.44%)	83 (45.86%)
Ciprofloxacin (CIP)	82 (72.57%)	77 (58.78%)	128 (70.72%)
Oxytetracycline (O)	92 (81.42%)	106 (80.92%)	125 (69.06%)

Out of 425 isolates, phenotypical production of β-lactamase enzymes was observed by double disc test in 141 (33.38%) isolates. Production of β-lactamase enzymes was detected maximum in *E. coli* (43.36%) isolates, followed by the *Salmonella* (27.48%) and *Klebsiella* (30.93%) strains (Table 1). The isolation rate of β-lactamase-producing *Enterobacteriaceae* was significantly higher (*p* < 0.05) in the birds reared in the South Andaman district than in the Nicobar district (Table 1). Using the cefoxitin-cloxacillin double disc synergy (CC-DDS) test, phenotypical AmpC production was found in 28.24% (120/425) bacterial isolates. *Klebsiella* (51.33%) was the highest AmpC producer, followed by *Salmonella* (36.28%) and *E. coli* (18.58%).

**Figure 2:**
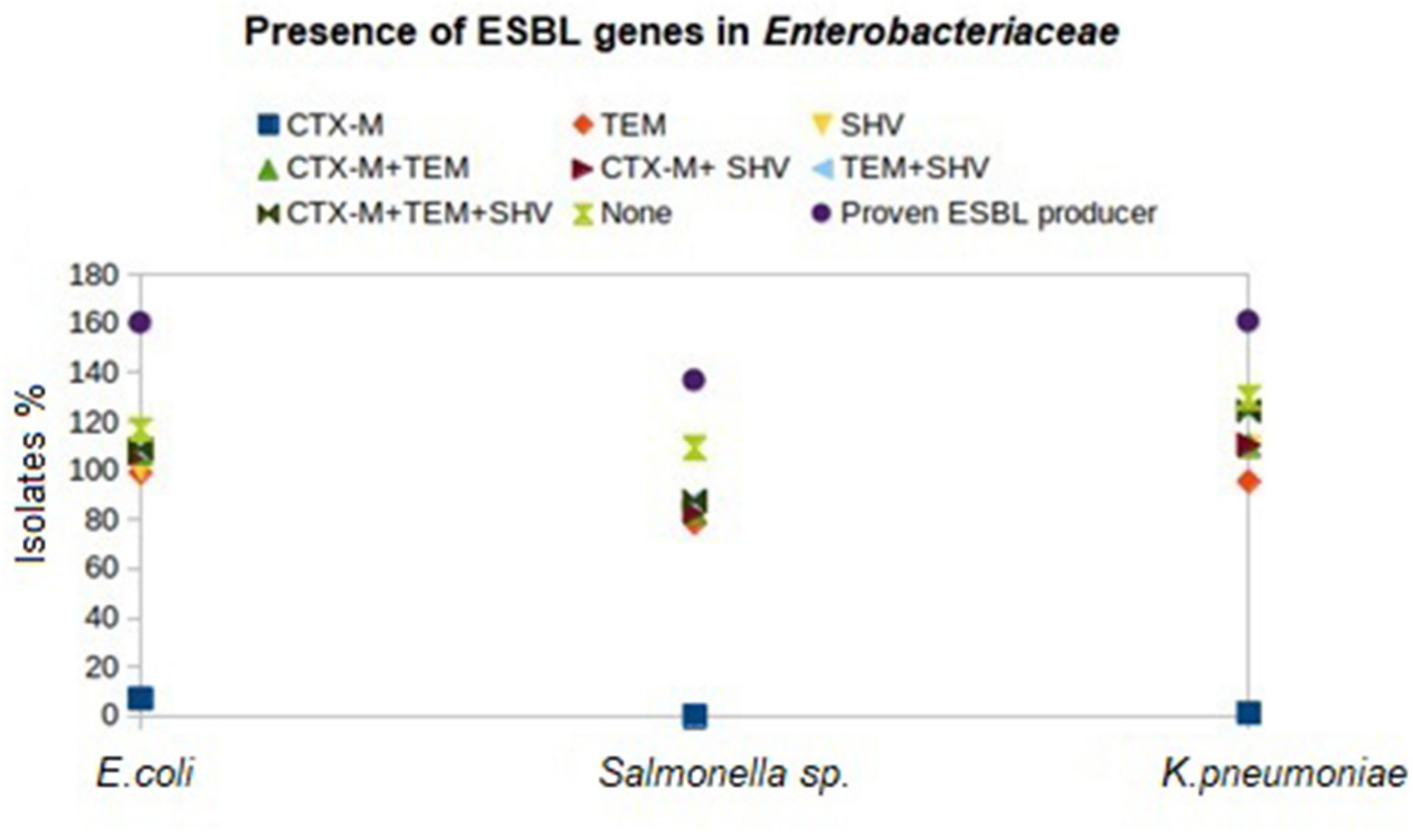
Distribution of β-lactamase genes in different birds reared in Andaman and Nicobar Islands (India).

Genotyping of the β-lactamase-producing isolates revealed maximum possession of *bla*_TEM_ by *E. coli* (92.04%), *Salmonella* (78.63%), and *Klebsiella* (94.48%) isolates followed by *bla*_SHV_ and *bla*_CTX − M_ (Figure 2). None of the *Salmonella* isolates possessed *bla*_CTX − M_. Moreover, none of the *E. coli, Salmonella*, and *Klebsiella* isolates possessed all the studied ESBL genes (*bla*_CTX − M_, *bla*_TEM_, and *bla*_SHV_) together. Furthermore, *bla*_TEM_ + *bla*_SHV_ genotype was possessed by the maximum number of isolates, followed by the genotype *bla*_TEM_ + *bla*_CTX − M_. All the phenotypical AmpC-producing isolates possessed *bla*_AmpC_ in PCR. The nucleotide sequences of the PCR products were compared and found similar with *bla*_CTX − M−15_ (98.1% cognate), *bla*_SHV − 11_ (99.45% cognate), *bla*_SHV − 27_ (98.01–99.38% cognate), *bla*_SHV − 228_ (99.38% cognate), *bla*_TEM − 1_ (99.33% cognate), and *bla*_AmpC_ (99.88% cognate) in the BLAST search. The sequences were published by DDBJ with accession numbers (https://getentry.ddbj.nig.ac.jp/) (Table 3).

**Table 3:** Accession numbers of nucleotide sequences of ESBL/AmpC genes possessed by *E. coli, Salmonella enterica*, and *Klebsiella pneumoniae* strains isolated from different birds in Andaman and Nicobar Islands (India).

**Bacteria**	**ESBL type**	**Source**	**Strain no**	**Place**	**Accession number**
*E. coli*	SHV-11	Desi bird	DPDB15	Diglipur, N&M/Andaman	LC655953
*E. coli*	TEM-1	Desi bird	BEBDB8	Beodnabad, S/Andaman	LC659951
*E. coli*	TEM-1	Layer	TBLB4	Terylabad, S/Andaman	LC659952
*E. coli*	TEM-1	Vanraja	BEBVR8	Beodnabad, S/Andaman	LC659953
*E. coli*	TEM-1	Vanraja	RGVR8	Nimbudera, N&M/Andaman	LC659954
*E. coli*	TEM-1	Nicobari	BLNB39	Big Lapathy, Nicobar	LC659955
*E. coli*	TEM-1	Vanraja	RGVR6	Nimbudera, N&M/Andaman	LC659960
*E. coli*	CTX-M-15	Nicobari	MPNB15	Manpur, S/Andaman	LC660645
*E. coli*	CTX-M-15	Desi bird	BEBDB8	Beodnabad, S/Andaman	LC660646
*E. coli*	CTX-M-15	Nicobari	SLNB30	Small Lapathy, Nicobar	LC660647
*E. coli*	AmpC	Desi bird	BEBDB5	Beodnabad, S/Andaman	LC661855
*E. coli*	AmpC	Desi bird	RGDB7	Rangat, N&M/Andaman	LC661856
*E. coli*	AmpC	Desi bird	DPDB21	Diglipur, N&M/Andaman	LC661857
*E. coli*	AmpC	Vanraja	RGVR5	Nimbudera, N&M/Andaman	LC661858
*E. coli*	AmpC	Vanraja	RGVR7	Nimbudera, N&M/Andaman	LC661859
*E. coli*	AmpC	Nicobari	KYKNB10	Kinyuka, Nicobar	LC661860
*E. coli*	AmpC	Nicobari	BLNB39	Big Lapathy, Nicobar	LC661861
*S. enterica*	SHV-228	Broiler	CABR1	Calicut, S/Andaman	LC656726
*S. enterica*	SHV-228	Nicobari	AHNB8	Dollygunj, S/Andaman	LC656727
*S. enterica*	TEM-1	Layer	AHLB9	Dollygunj, S/Andaman	LC656923
*S. enterica*	AmpC	Broiler	INBR28	Indiranagar, S/Andaman	LC661874
*S. enterica*	AmpC	Desi bird	KGDB21	Kodiyaghat, S/Andaman	LC661875
*S. enterica*	AmpC	Vanraja	BEBVR7	Beodnabad, S/Andaman	LC661876
*S. enterica*	AmpC	Layer	AHLB10	Dollygunj, S/Andaman	LC661877
*S. enterica*	AmpC	Desi bird	DPDB11	Diglipur, N&M/Andaman	LC661878
*S. enterica*	AmpC	Vanraja	RGVR8	Nimbudera, N&M/Andaman	LC661879
*K. pneumoniae*	SHV-27	Desi bird	BTDB29	Baratang, N&M/Andaman	LC653140
*K. pneumoniae*	SHV-11	Nicobari	KYKNB10	Kinyuka, Nicobar	LC655875
*K. pneumoniae*	TEM-1	Layer	TBLB2	Terylabad, S/Andaman	LC659956
*K. pneumoniae*	TEM-1	Nicobari	KGNB4	Kodiyaghat, S/Andaman	LC659957
*K. pneumoniae*	TEM-1	Desi bird	RCDB16	Rangachang, S/Andaman	LC659958
*K. pneumoniae*	TEM-1	Desi bird	DPDB20	Diglipur, N&M/Andaman	LC659959
*K. pneumoniae*	TEM-1	Nicobari	BLNB33	Big Lapathy, Nicobar	LC659961
*K. pneumoniae*	TEM-1	Nicobari	TLNB45	Tamaloo, Nicobar	LC659962
*K. pneumoniae*	TEM-1	Vanraja	LPVR16	LalPahad, S/Andaman	LC659963
*K. pneumoniae*	TEM-1	Vanraja	DPVR14	Diglipur, N&M/Andaman	LC659964
*K. pneumoniae*	CTX-M-15	Desi bird	DPDB19	Diglipur, N&M/Andaman	LC660643
*K. pneumoniae*	CTX-M-15	Desi bird	DPDB22	Diglipur, N&M/Andaman	LC660644
*K. pneumoniae*	AmpC	Nicobari	PKNB15	Perka,C/N	LC661862
*K. pneumoniae*	AmpC	Nicobari	BLNB33	Big Lapathy, Nicobar	LC661863
*K. pneumoniae*	AmpC	Nicobari	TLNB45	Tamaloo, Nicobar	LC661864
*K. pneumoniae*	AmpC	Broiler	MPBR9	MaccaPahad, S/Andaman	LC661865
*K. pneumoniae*	AmpC	Layer	AHLB8	Dollygunj, S/Andaman	LC661866
*K. pneumoniae*	AmpC	Nicobari	KGNB4	Kodiyaghat, S/Andaman	LC661867
*K. pneumoniae*	AmpC	Desi bird	BEBDB6	Beodnabad, S/Andaman	LC661868
*K. pneumoniae*	AmpC	Vanraja	LPVR17	LalPahad, S/Andaman	LC661869
*K. pneumoniae*	AmpC	Desi bird	RGDB6	Rangat, N&M/Andaman	LC661870
*K. pneumoniae*	AmpC	Desi bird	DPDB20	Diglipur, N&M/Andaman	LC661871
*K. pneumoniae*	AmpC	Vanraja	RGVR6	Nimbudera, N&M/Andaman	LC661872
*K. pneumoniae*	AmpC	Vanraja	DPVR14	Diglipur, N&M/Andaman	LC661873

**Figure 3:**
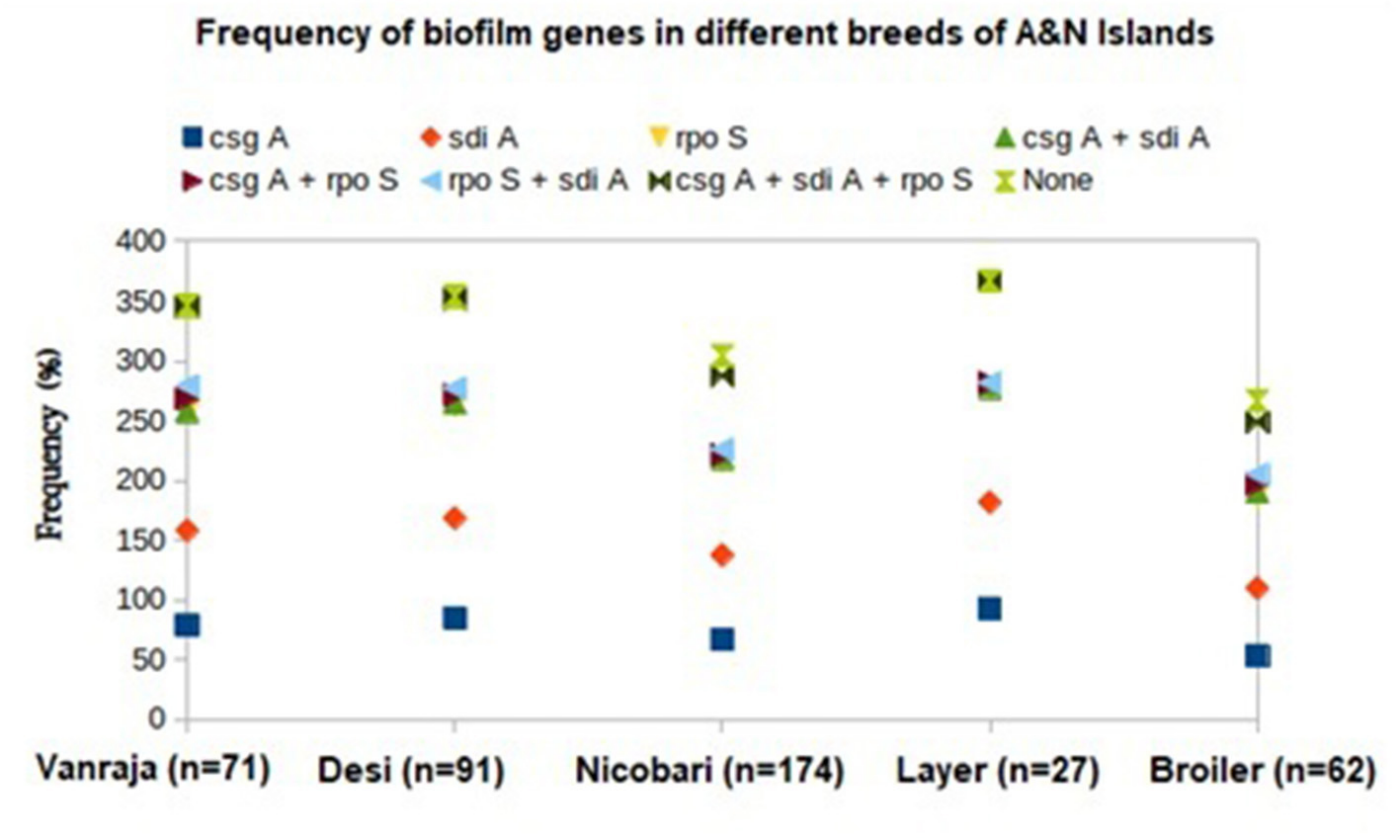
Distribution of biofilm-associated genes in different birds reared in Andaman and Nicobar Islands (India).

The distribution of biofilm-associated genes (*csgA, rpoS*, and *sdiA*) in the studied *Enterobacteriaceae* strains from different breeds or varieties of the birds revealed the maximum possession mostly by layer birds, followed by the other varieties of the studied birds (Figure 3). The *csgA* was detected with the highest frequency in the isolates from layer birds (92.5%), followed by *Desi* (84.6%), *Vanraja* (79%), *Nicobari* (66.6%), and broiler (53.2%). The *sdiA* was detected with the highest frequency in the isolates from layer birds (88.8%), followed by *Desi* (83.5%), *Vanraja* (79%), *Nicobari* (70.6%), and broiler (56.4%). The *rpoS* was detected with the highest frequency in the isolates from *Vanraja* (99%), followed by layer birds (96.3%), *Desi* (94.5%), *Nicobari* (78.7%), and broiler (77.4%).

**Figure 4:**
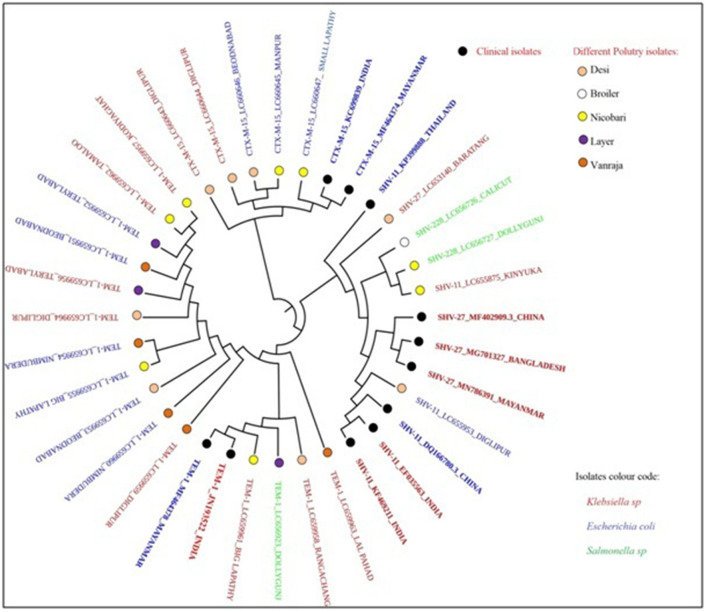
Clonal relationship of β-lactamase-producing *Enterobacteriaceae* isolated from locally reared fowls in Andaman and Nicobar Islands (India) with human clinical isolates.

The phylogenetic analysis revealed a partial clonal relationship of β-lactamase sequences of the present study (Table 3), i.e., 15 *bla*_TEM − 1_ (LC659951-64 and LC656923), 2 *bla*_SHV − 228_ (LC656726-27), 5 *bla*_CTX − M−15_ (LC660643-47), 1 *bla*_SHV − 27_ (LC653140), and 2 *bla*_SHV − 11_ (LC655875 and LC655953) sequences with *bla*_CTX − M−15_, *bla*_SHV − 11_, and *bla*_SHV − 27_ and *bla*_TEM − 1_ sequences possessed by clinical strains isolated from human patients in India, Bangladesh, China, Myanmar, and Thailand (Figure 4).

**Figure 5:**
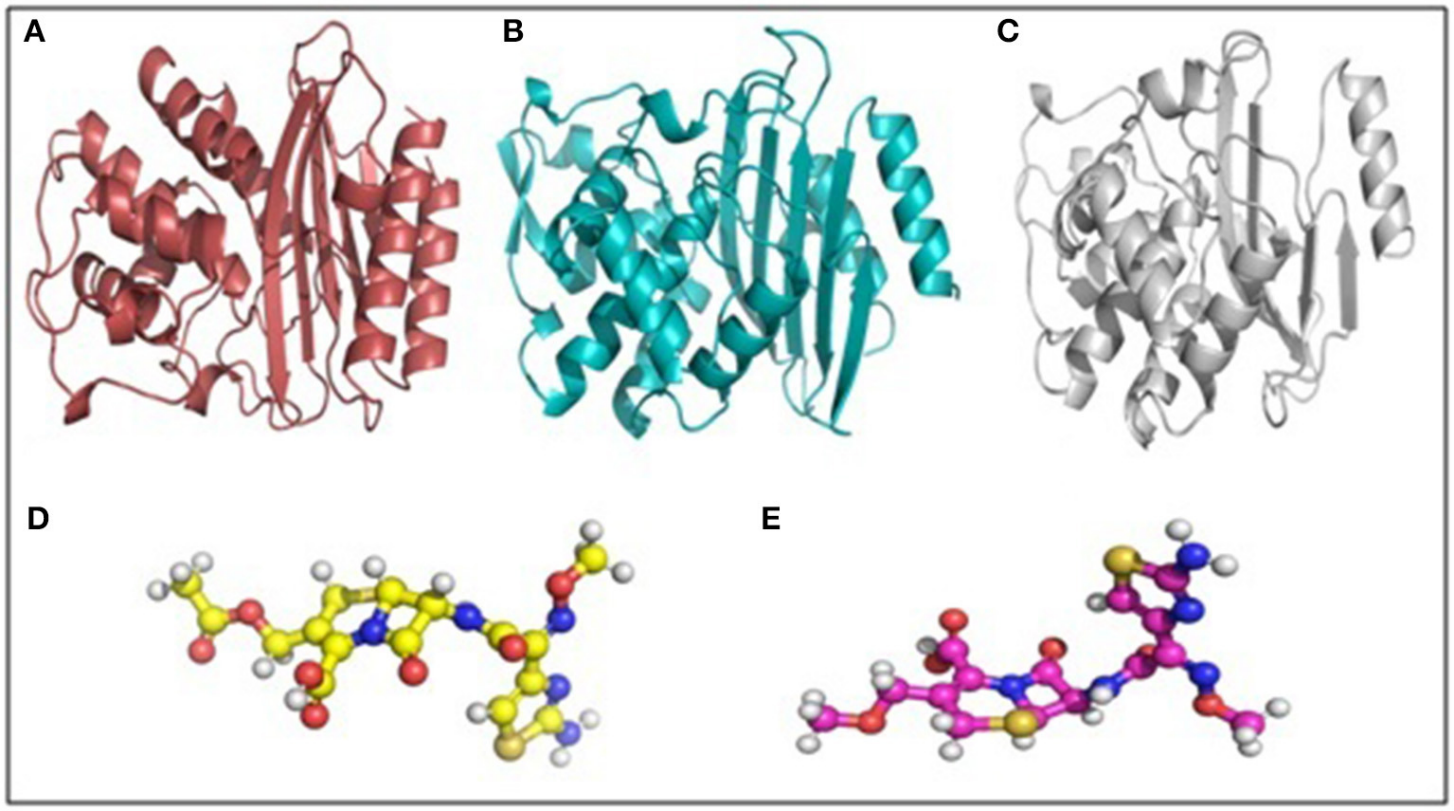
Macromolecule and ligand representation (3D) in PyMOL. These color codes have been mentioned throughout the study. **(A)** Cartoon representation of TEM-1 in ruby color. **(B)** Cartoon representation of CTX-M-15 in cyan color. **(C)** Cartoon representation of SHV in gray color. **(D)** Ball and stick representation of cefotaxime (ligand bonds are in golden color). **(E)** Ball and stick representation of cefpodoxime (ligand bonds are in lilac color).

**Figure 6:**
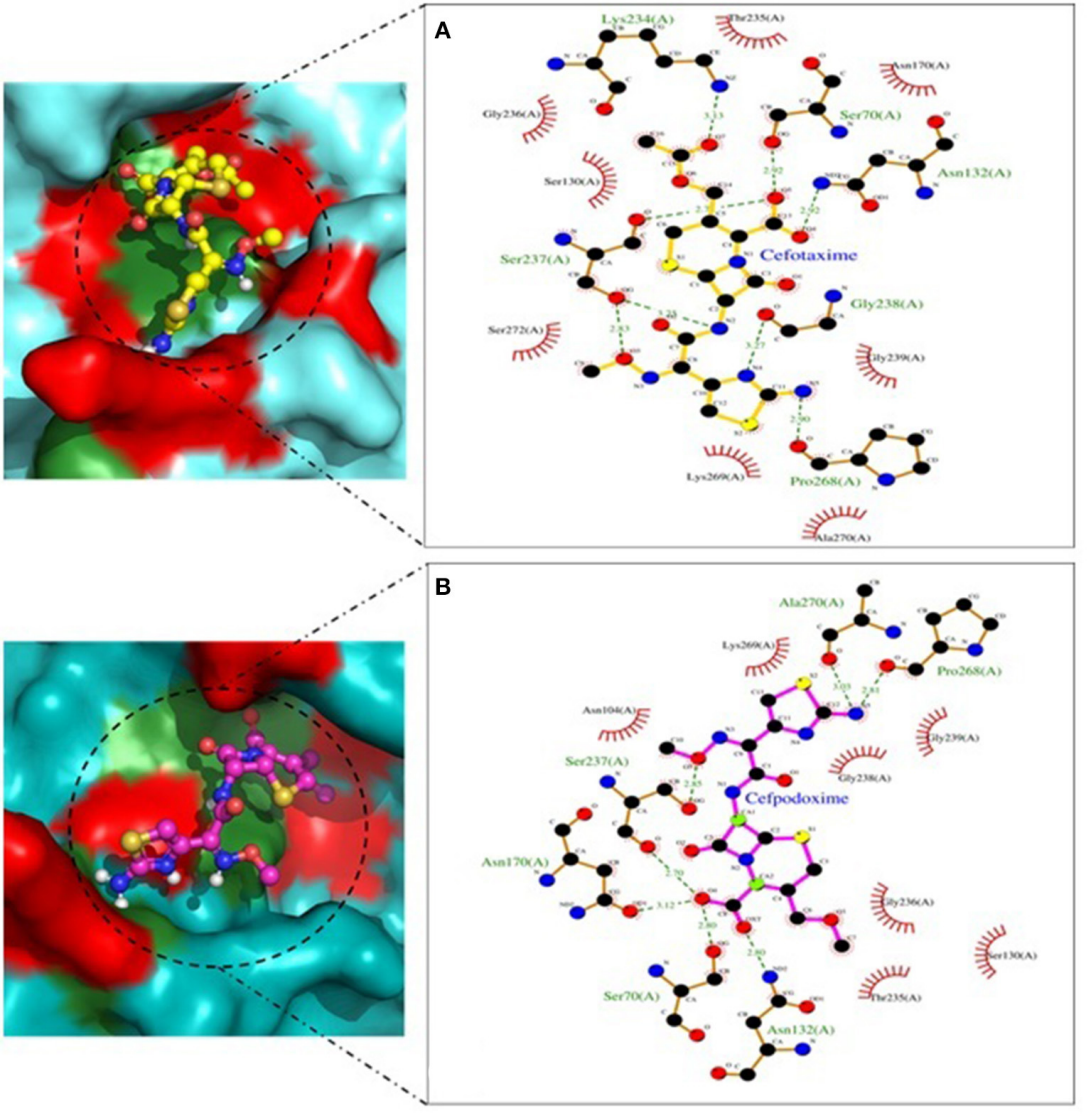
CTX-M-15 involved in interactions. **(A)** A binding map for cefotaxime+CTX-M-15. **(B)** A binding map for cefpodoxime+CTX-M-15.

**Figure 7:**
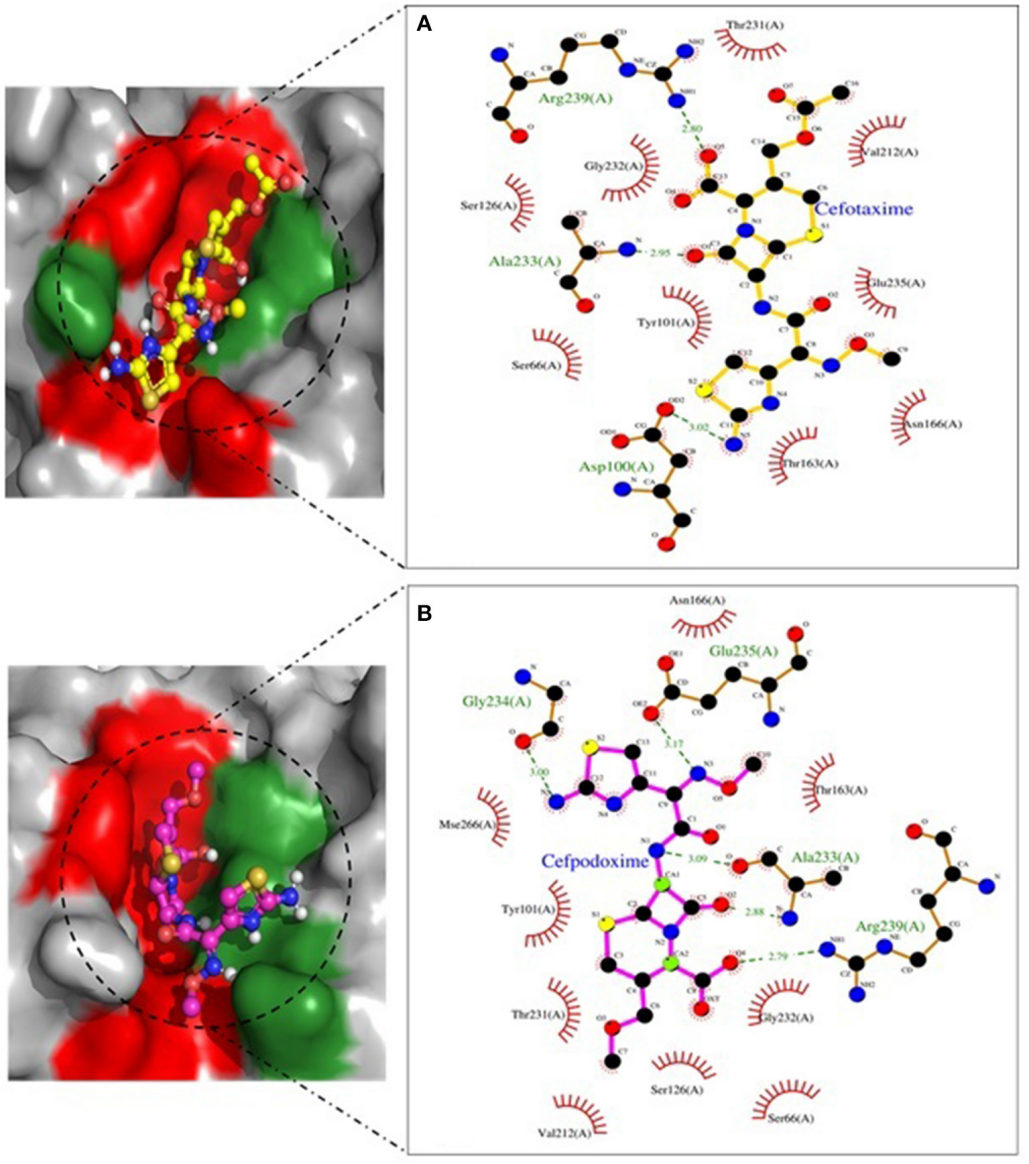
SHV-11 involved in interactions. **(A)** A binding map for cefotaxime+SHV-11. **(B)** A binding map for cefpodoxime+SHV-11.

**Figure 8:**
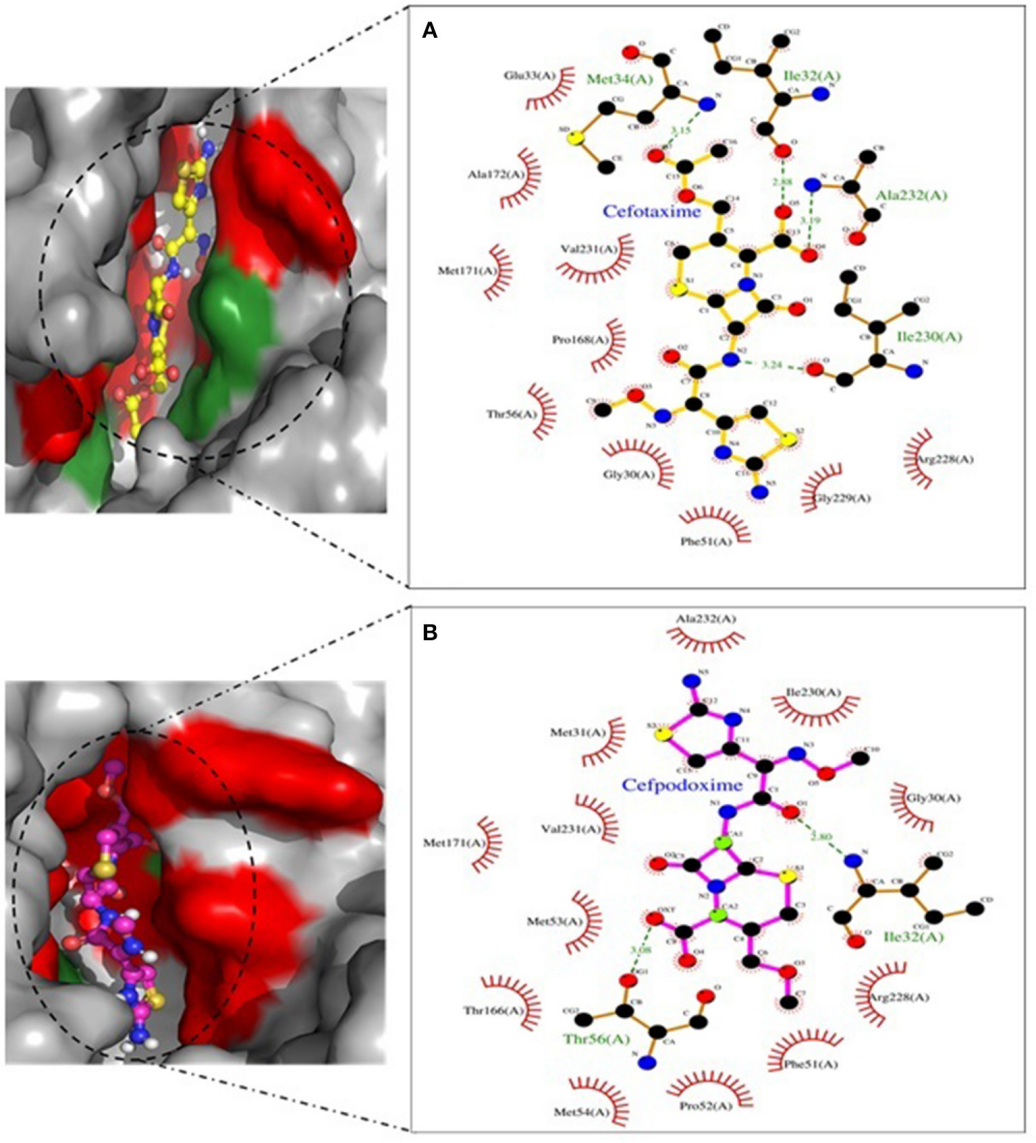
SHV-27 involved in interactions. **(A)** A binding map for cefotaxime+SHV-27. **(B)** A binding map for cefpodoxime+SHV-27.

**Figure 9:**
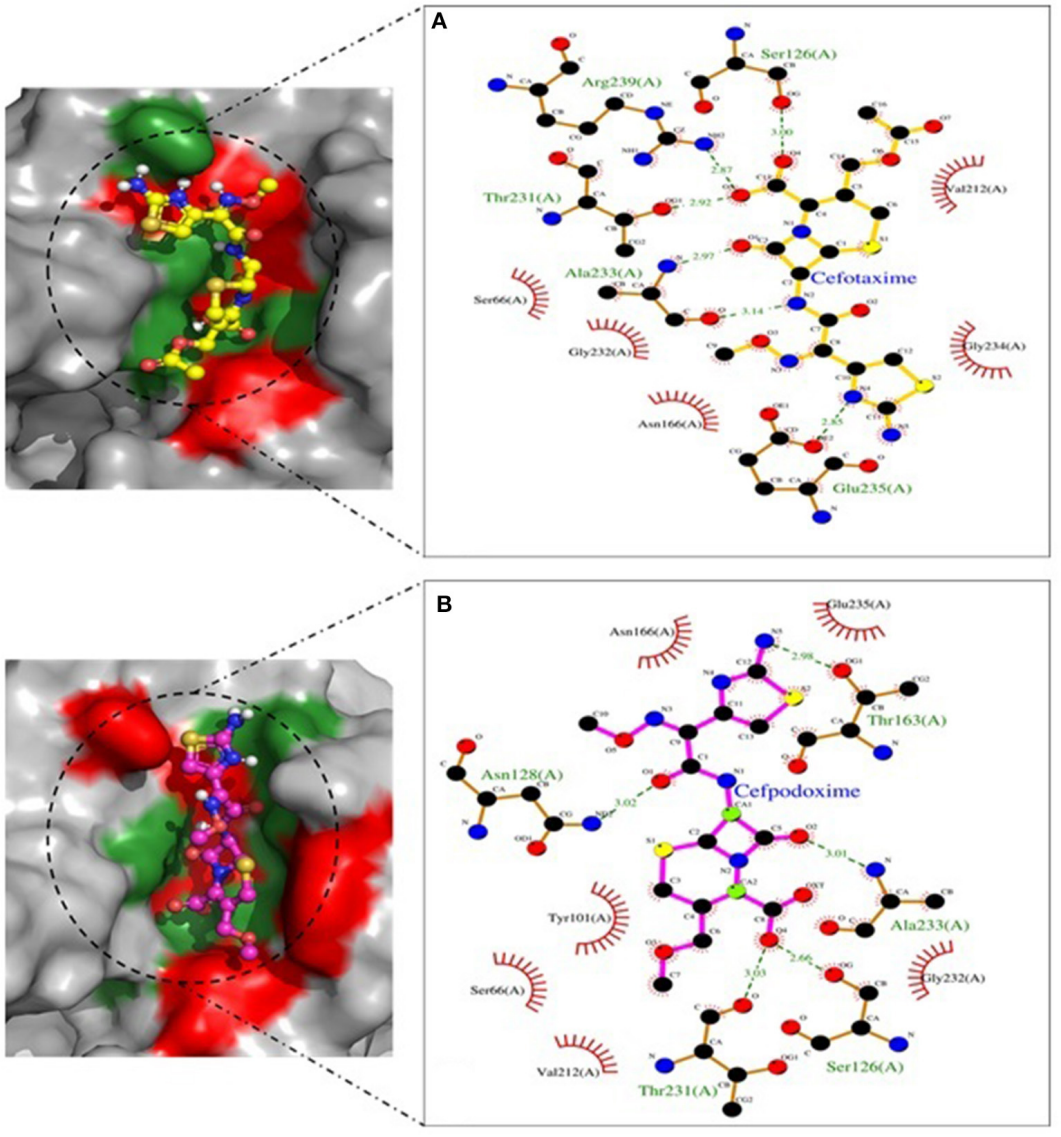
SHV-228 involved in interactions. **(A)** A binding map for cefotaxime+SHV-228. **(B)** A binding map for cefpodoxime+SHV-228.

**Figure 10:**
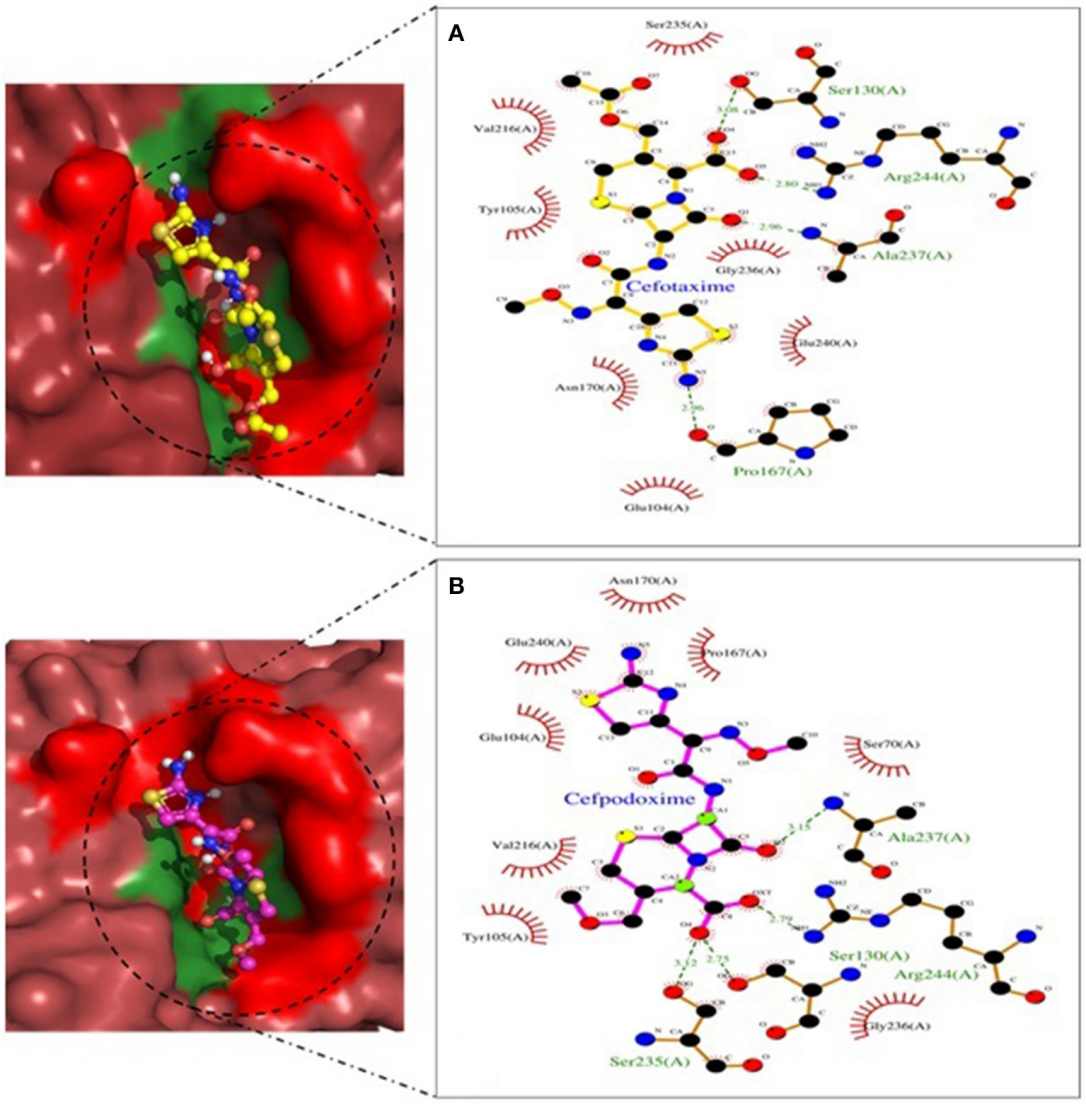
TEM-1 involved in interactions. **(A)** A binding map for cefotaxime+TEM-1. **(B)** A binding map for cefpodoxime+TEM-1.

Molecular docking depicted the Gibbs free energy release for 10 different macromolecules (proteins) and ligand (antibiotic) complexes, ranging from −8.1 (SHV-27+cefotaxime) to −7 (TEM-1+cefotaxime) kcal/mol. The color code of the drug and receptor molecules was maintained throughout the study (Figure 5). A summary of all the 10 complexes and participating amino acid residues in molecular interaction is described in Table 4. Different ligand + receptor complexes (2D Ligplot Plus and 3D PyMOL) are described in Figures 6–10.

**Table 4:** Summary of *in silico* analyses.

**Results of Autodock Vina**				**Amino acid residues** ^**¥**^	
**ESBLs (Protein) name**		**Ligand structure**	**Gibbs free energy (Kcal/mol)**	**Hydrogen bonding**	**Hydrophobic interactions**
**CTX-M-15**		**Cefotaxime**	**−7.5**	**Ser 70**, **Asn 132**, Lys 234, **Ser 237**, **Gly 238**, **Pro 268**	**Ser 130**, **Asn 170**, **Thr 235**, **Gly 236**, **Gly 239**, Ser 272, **Lys 269**, **Ala 270**
		**Cefpodoxime**	**−7.1**	**Ser 70**, **Asn 132**, **Asn 170**, **Ser 237**, **Ala 270**, **Pro 268**	Asn 104, **Ser 130**, **Thr 235**, **Gly 236**, **Gly 238**, **Gly 239**, **Lys 269**
**SHV**	**SHV-11**	**Cefotaxime**	**−7.8**	Asp 100, **Ala 233**, **Arg 239**	**Ser 66**, **Tyr 101**, **Ser 126**, **Thr 163**, **Asn 166**, **Val 212**, **Thr 231**, **Gly 232**, **Glu 235**
		**Cefpodoxime**	**−8.0**	**Ala 233**, Gly 234, **Glu 235**, **Arg 239**	**Ser 66**, **Tyr 101**, **Ser 126**, **Thr 163**, **Asn 166**, **Val 212**, **Thr 231**, **Gly 232**, Mse 266
	**SHV-27**	**Cefotaxime**	**−8.1**	**Ile 32**, Met 34, **Ile 230**, **Ala 232**	**Gly 30**, Glu 33, **Phe 51**, **Thr 56**, Pro 168, **Met 171**, Ala 172, **Arg 228**, Gly 229, **Val 231**
		**Cefpodoxime**	**−7.5**	**Ile 32**, **Thr 56**	**Gly 30**, Met 31, **Phe 51**, Pro 52, Met 53, Met 54, Thr 166, **Met 171**, **Arg 228**, **Ile 230**, **Val 231**, **Ala 232**
	**SHV-228**	**Cefotaxime**	**−7.3**	**Ser 126**, **Thr 231**, **Ala 233**, **Glu 235**, Arg 239	**Ser 66**, **Asn 166**, **Val 212**, **Gly 232**, Gly 234
		**Cefpodoxime**	**−7.1**	**Ser 126**, Asn 128, Thr 163, **Thr 231**, **Ala 233**	**Ser 66**, Tyr 101, **Asn 166**, **Val 212**, **Gly 232**, **Glu 235**
**TEM-1**		**Cefotaxime**	**−7**	**Ser 130**, **Pro 167**, **Ala 237**, **Arg 244**	**Glu 104**, **Tyr 105**, **Asn 170**, **Val 216**, **Ser 235**, **Gly 236**, **Glu 240**
		**Cefpodoxime**	**−7.3**	**Ser 130**, **Ser 235**, **Ala 237**, **Arg 244**	Ser 70, **Glu 104**, **Tyr 105**, **Pro 167**, **Asn 170**, **Val 216**, **Gly 236**, **Glu 240**

¥ amino acids, being reported necessary for these catalytic activities, are mentioned in bold.

## Discussions

In the present study, *K. pneumoniae* was found to be the most prevalent in the cloacal swabs of the birds collected from different districts of A&N Islands (India), followed by *Salmonella* (30.82%) and *E. coli* (26.58%). Similar isolation rates of *Klebsiella* were reported earlier from poultry (43.8–72.3%) and bovine milk samples (45.2%) in other parts of India (25, 26). The isolation rate of *Salmonella* and *E. coli* in the present study was found to be corroborative with earlier reports (27, 28). However, the recovery of *Salmonella, E. coli*, and *Klebsiella* from poultry varied in different geographical regions depending on isolation protocol, sample size, and animal husbandry practices (29).

Antibiogram profiling of all 425 isolates showing maximum resistance against tetracycline is corroborative with the previous findings in Bangladesh (30), Iran (31), Malaysia (32), and Egypt (33). The resistance of poultry origin*-Enterobacteriaceae* to quinolone antibiotics (ciprofloxacin) from South China (34), Spain (35), and Egypt (36) was also reported, where co-resistance to ciprofloxacin and tigecycline was reported. Resistance to quinolones is often linked to tetracycline as the tetracycline molecule activates mutations in the *mar* operon, which results in more expression of the MarA protein increasing multidrug resistance (37). Most of the isolates in the present study showed resistance to three or more antibiotics and were considered multidrug resistant (38). The most common MDR pattern was found as E-TE-C-AMC-SF-COT-AMP-AX-CIP-O (9.7% in *E. coli*, 6.1% in *Salmonella*, and 5% in *Klebsiella*). All three studied bacterial strains (86.19%) were found susceptible to gentamicin, indicating the possible future usage of gentamicin for the treatment of bacterial infections in poultry in the A&N Islands.

Phenotypical β-lactamase production was detected maximum in *E. coli* (43.36%) isolates, followed by *Salmonella* (27.48%) and *Klebsiella* (30.93%). The majority of the ESBL producers in poultry belonged to the *E. coli* and *Salmonella* group of bacteria throughout the world (7). The occurrence of ESBL-producing *Enterobacteriaceae* was in accordance with those reported in Thailand (24.9%) (39), Lebanon (28%) (29), Ghana (29%) (4), and Denmark (27%) (40), lower than the prevalence rate reported in Germany (81–85%) (41) and Spain (79%) (42), and higher than Nicaragua (13%) (43) and Finland (14%) (44). The occurrence of ESBL-producing *Enterobacteriaceae* in poultry varies widely according to geographical location and antibiotic exposure, and the plasmids play a significant role in the clonal spread of ESBL genes in the poultry production system as the vertical route has less importance (44).

The isolation rate of β-lactamase producing *Enterobacteriaceae* was significantly higher (*p* < 0.05) in the birds reared in the South Andaman district than in Nicobar, which is correlated with more anthropogenic activities as the total human population and population density of South Andaman is significantly higher than the Nicobar (45). Anthropogenic activities were found to be directly correlated with the generation of ESBL-resistome in the environment either due to the dissemination of ESBL-producing bacteria or the release of the antimicrobials at the sub-therapeutic level in the environment (8–10). However, the occurrence of β-lactamase-producing *Enterobacteriaceae* in the birds reared in the Nicobar Islands with the minimum anthropogenic activities is an important finding as it may be correlated with increased soil salinity and high incidence of migratory birds in the islands after tsunami (46). Increased translation of multiple antibiotic resistance operons and transfer of ESBL gene containing plasmid was detected in soil bacteria to cope with the salinity stress as the stressors and the antimicrobials use the same bacterial cellular components or processes (47, 48). An increased presence of migratory birds after tsunami was associated with the generation of feeding habitats by the submergence of agricultural fields (49).

The nucleotide sequencing of the PCR products revealed that the variants of the β-lactamase circulating in the fowl population of A&N Islands were TEM-1 with the highest frequency, followed by CTX-M-15, SHV-11, SHV-27, and SHV-228. Similarly, TEM-1 was reported with a maximum frequency in *E. coli* strains isolated from diseased poultry in China (50) and in *Salmonella* strains isolated from poultry or poultry products in the Netherlands (51). Although TEM-1 is not considered as a classical ESBL, it is reported with high frequency in human clinical isolates throughout the world, and TEM-1-encoded enzyme was sometimes detected to demonstrate ESBL properties (52, 53). The high prevalence of TEM-1 in the fowl population of the present study also indicated the probable presence of subclinical bacterial infections, which was overlooked by the farmers who were not trained in poultry farming (13). The possession of *bla*_CTX − M−15_ is mostly associated with clinical *Enterobacteriaceae* isolates originated from both human and animal populations worldwide (54). CTX-M-15-producing *Enterobacteriaceae* were earlier reported in poultry from different parts of the globe (41, 42, 55). The SHV-27 was earlier reported in *Klebsiella* strains isolated from neonatal blood in Brazil, and the enzyme was found to show resistance against cefotaxime, ceftazidime and aztreonam (56). However, SHV-27, SHV-11, and SHV-228 were not reported from poultry in any part of the world.

Using the cefoxitin-cloxacillin double disc synergy (CC-DDS) test, phenotypical AmpC enzyme production was found to be 28.24% (120/425). In India, earlier studies revealed the occurrence of chromosomal AmpC (*bla*_AmpC_) in *Enterobacteriaceae* strains isolated from poultry, cattle with mastitis, pig and farm environments, and ducks (57). Other than therapeutic exposure to cefotaxime and ceftazidime, which was not detected in the present study, the occurrence of AmpC-producing bacteria might be associated with clonal transmission from the environment, as observed in a transmission dynamics study of ESBL-producing *Enterobacteriaceae* (39). The co-existence of ESBL and AmpC enzymes was detected in 10.82% (46/425) of the isolates, which is consistent with the earlier findings in the poultry production system (29, 58).

The generation of environmental resistomes is dependent on the persistence of ESBL/AmpC-producers on the abiotic or biotic surface with the capacity to form biofilms, as it helps in the survival of the bacterial colony against physical and chemical stresses (11). The present study detected a high prevalence (76%) of biofilm-associated genes in the *Enterobacteriaceae* strains isolated from the studied fowl population, indicating their possible environmental origin, although the soil microbial profile and the phenotypical biofilm-forming capacity of the strains were not validated.

The phylogenetic analysis revealed a partial clonal relationship between the fowl origin *Enterobacteriaceae* isolates and human clinical strains from the Indian subcontinent. Earlier studies revealed genetic relatedness of strains, similarity in types of β-lactamase genes, and/or associated plasmids in *E. coli* strains originating from animals and humans depicting the transmission probabilities (59).

Molecular docking interaction in the present study demonstrated the probable interactions among the different macromolecule-ligand complexes. The ligands with the minimum binding energy have the highest affinity of β-lactamases for cefotaxime and cefpodoxime. In our study, SHV-27 variants possessed the highest activity against cefotaxime. Improved docking scores were observed for the SHV variants because of the size and volume of its catalytic pocket and its druggability (Supplementary Figure 2). Antibiotic degradation by *bla*_SHV_ in the present study has also revealed the participation of almost equivalent amino acids in terms of hydrophobic contacts (Ser 66, Tyr 101, Asn 166, and Val 212), which further emphasizes the structural homology of the other related variants. The study suffers from limitations related to sequencing, clonality analysis, and restricted numbers of isolates. The future characterization of this geographical location with the advent of next-generation sequencing can reveal the picture in detail.

The present study thus described the occurrence of β-lactamase/AmpC-producing *Enterobacteriaceae* in the local fowl population, even with the exposure of limited anthropogenic activities. Most of the strains possessed *bla*_TEM − 1_, followed by *bla*_CTX − M−15_. The possession of *bla*_SHV − 11_, *bla*_SHV − 27_, and *bla*_SHV − 228_ in poultry *Enterobacteriaceae* strains was not reported earlier. ESBL variants were modeled by the SWISS-MODEL and verified. Ligand with the minimum binding energy has the highest affinity of β-lactamases for cefotaxime and cefpodoxime. Phylogenetic analysis of the fowl origin ESBL-producing *Enterobacteriaceae* strains revealed a partial clonal relationship with the clinical isolates from human patients.

## Data availability statement

The datasets presented in this study can be found in online repositories. The names of the repository/repositories and accession number(s) can be found in the article/Supplementary material.

## Ethics statement

The animal study was reviewed and approved by Institutional Animal Ethics Committee, WBUAFS. Written informed consent was obtained from the owners for the participation of their animals in this study.

## Author contributions

SBh collected the samples and did all the laboratory works. SP conducted bioinformatics analysis. JS and IS supervised the study. TS, AD, SJ, KB, TD, SBa, and AT conceptualized the study. TM and AS helped in the analysis. IS, SBh, and AT wrote the primary and revised manuscripts. All authors contributed to the article and approved the submitted version.

## Supplementary material

Supplementary Figure 1 RC plot analyses for the modeled SHV-27 protein structure.

Supplementary Figure 2 Summary of main chain parameters for the modeled SHV-27 protein structure.

Supplementary Figure 3 Summary of side chain parameters for the modeled SHV-27 protein structure.

Supplementary Figure 4 RMSD profile for the modeled SHV-27 protein structure.

Supplementary Figure 5 RC plot analyses for the modeled SHV-228 protein structure.

Supplementary Figure 6 Summary of main chain parameters for the modeled SHV-228 protein structure.

Supplementary Figure 7 Summary of side chain parameters for the modeled SHV-228 protein structure.

Supplementary Figure 8 RMSD profile for the modeled SHV-228 protein structure.

Supplementary Figure 9 Surface view of macromolecules. **(A)** TEM-1 (in ruby color) showing the opening of the catalytic pocket marked in yellow color. **(B)** CTX-M-15 (in cyan color) showing the opening of the catalytic pocket marked in yellow color. **(C)** SHV (in gray color) showing the opening of the catalytic pocket marked in yellow color.
