# Supplementary figures and images for: Exploring broilers and native fowls of Andaman and Nicobar Islands as a source of β-lactamase-producing Enterobacteriaceae even with limited anthropogenic activities and docking-based identification of catalytic domains in novel β-lactamase variants
Source: Front Vet Sci. 2023 Jan 5;9:1075133. doi: 10.3389/fvets.2022.1075133 (PMC9849777; doi:10.3389/fvets.2022.1075133)

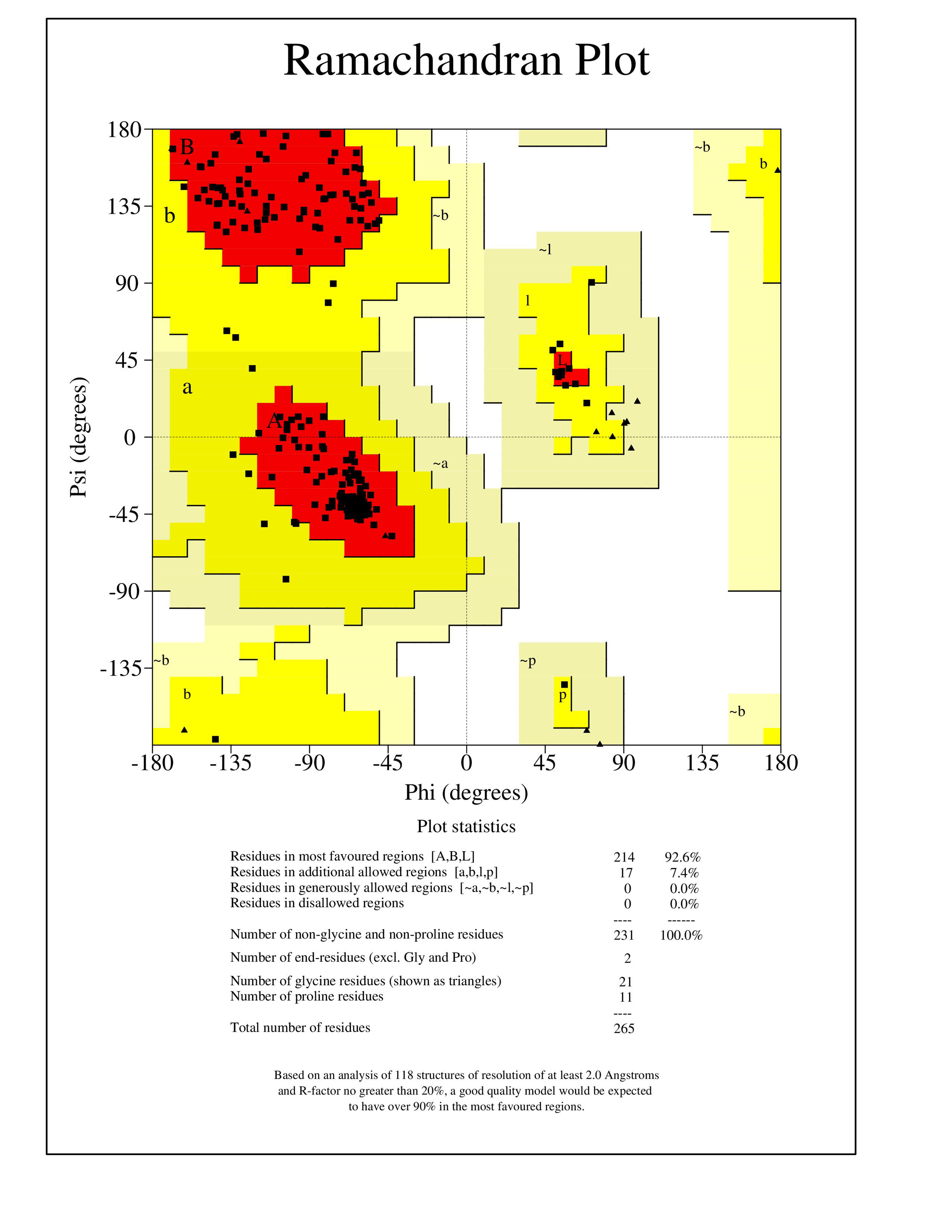

Supplement: Supplementary Figure 1 — RC plot analyses for the modeled SHV-27 protein structure. [file Image_1.JPEG]

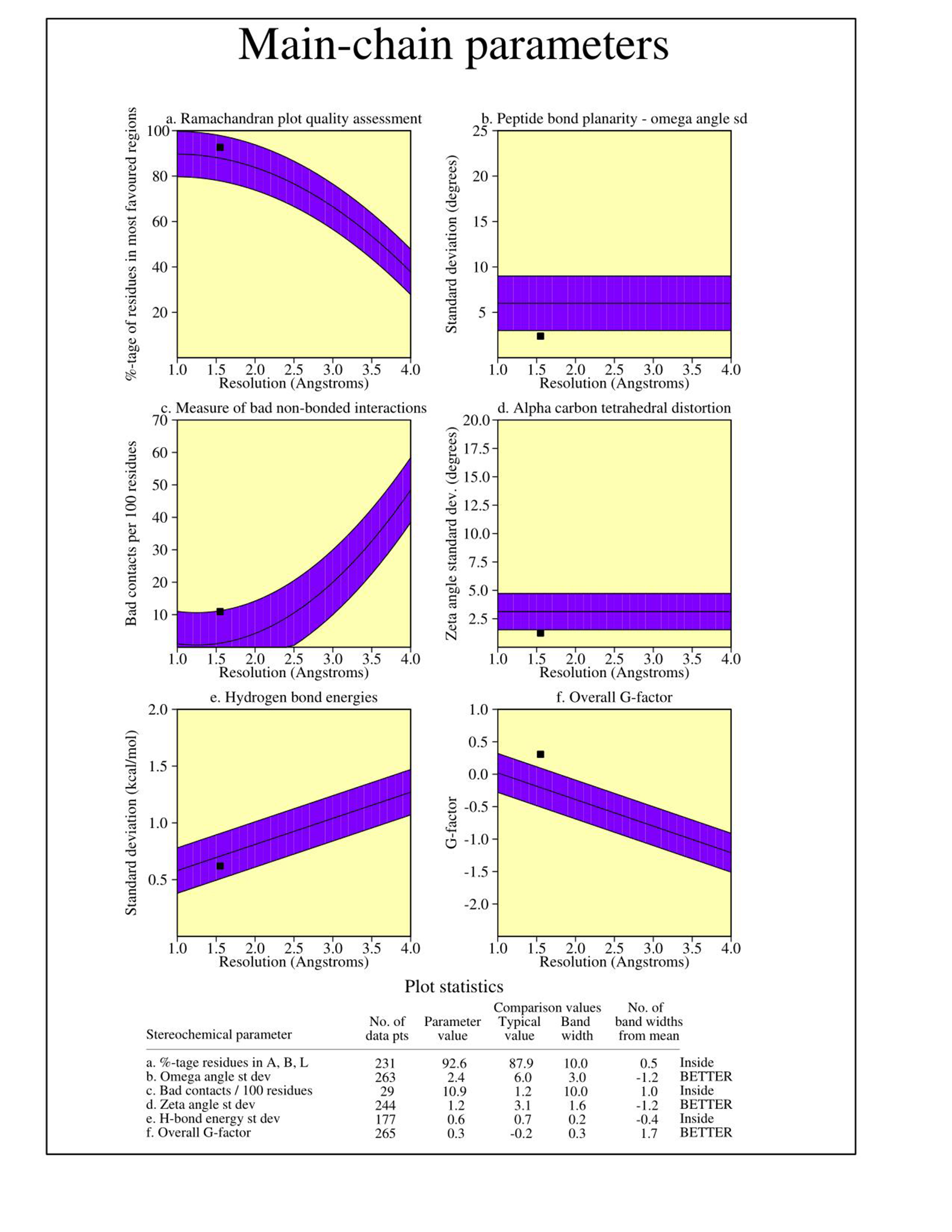

Supplement: Supplementary Figure 2 — Summary of main chain parameters for the modeled SHV-27 protein structure. [file Image_2.JPEG]

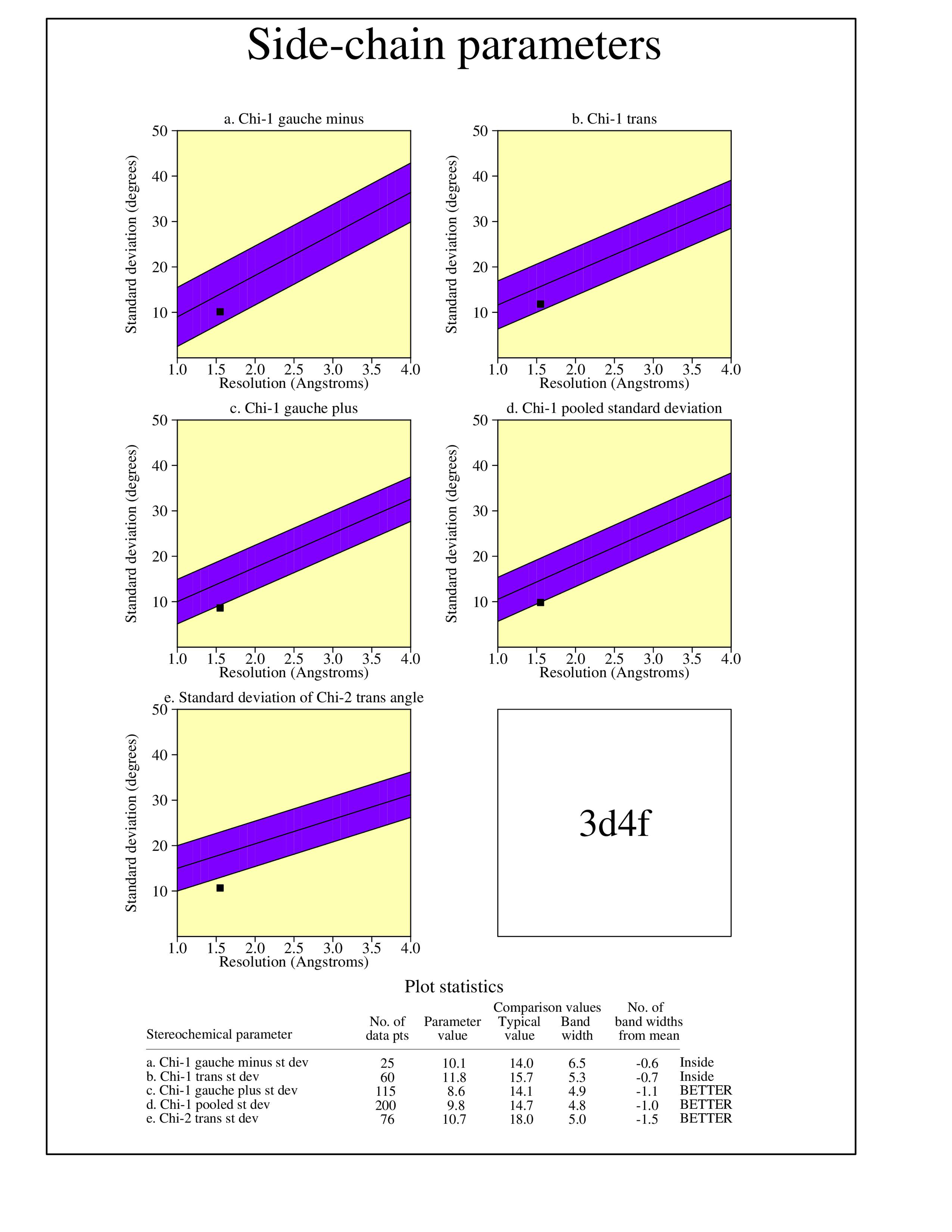

Supplement: Supplementary Figure 3 — Summary of side chain parameters for the modeled SHV-27 protein structure. [file Image_3.JPEG]

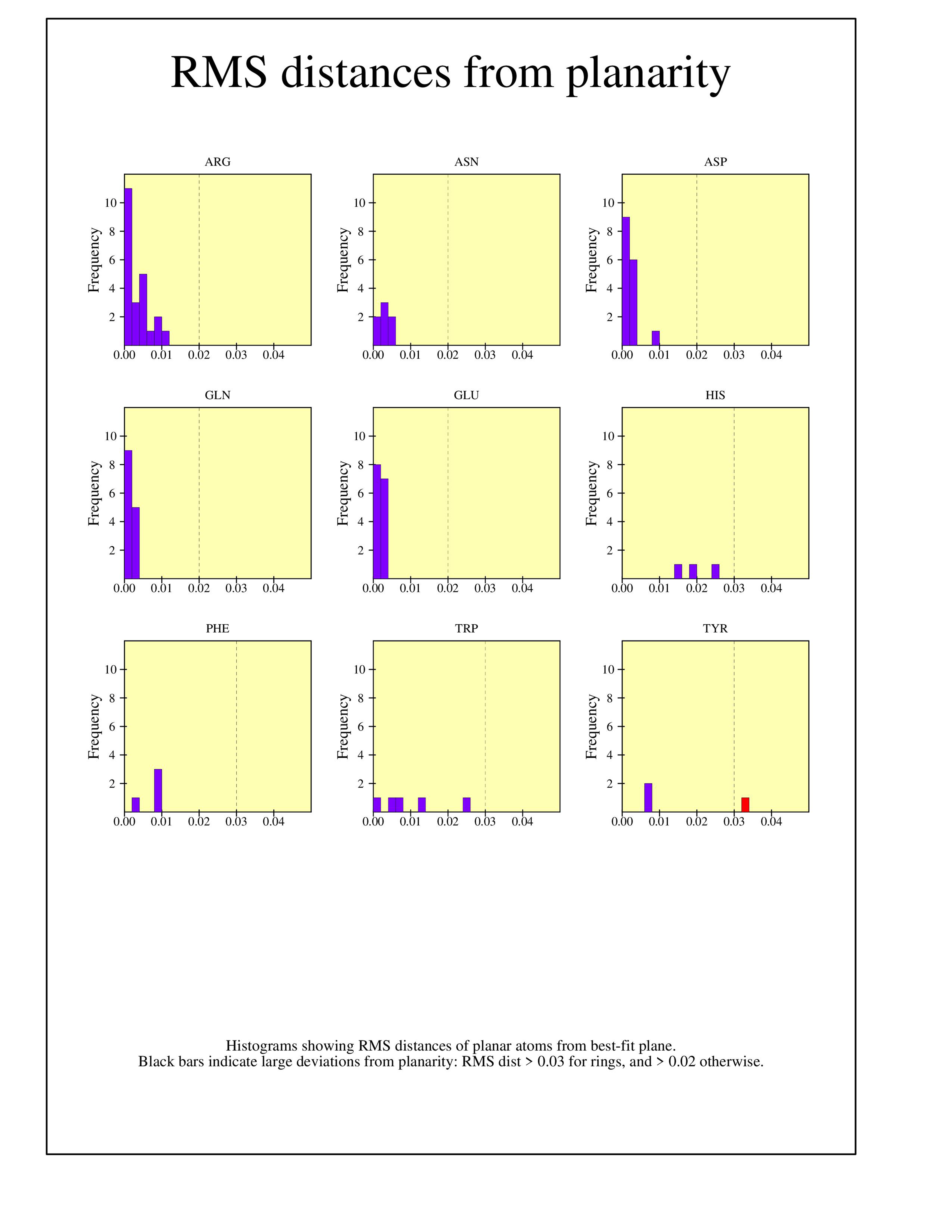

Supplement: Supplementary Figure 4 — RMSD profile for the modeled SHV-27 protein structure. [file Image_4.JPEG]

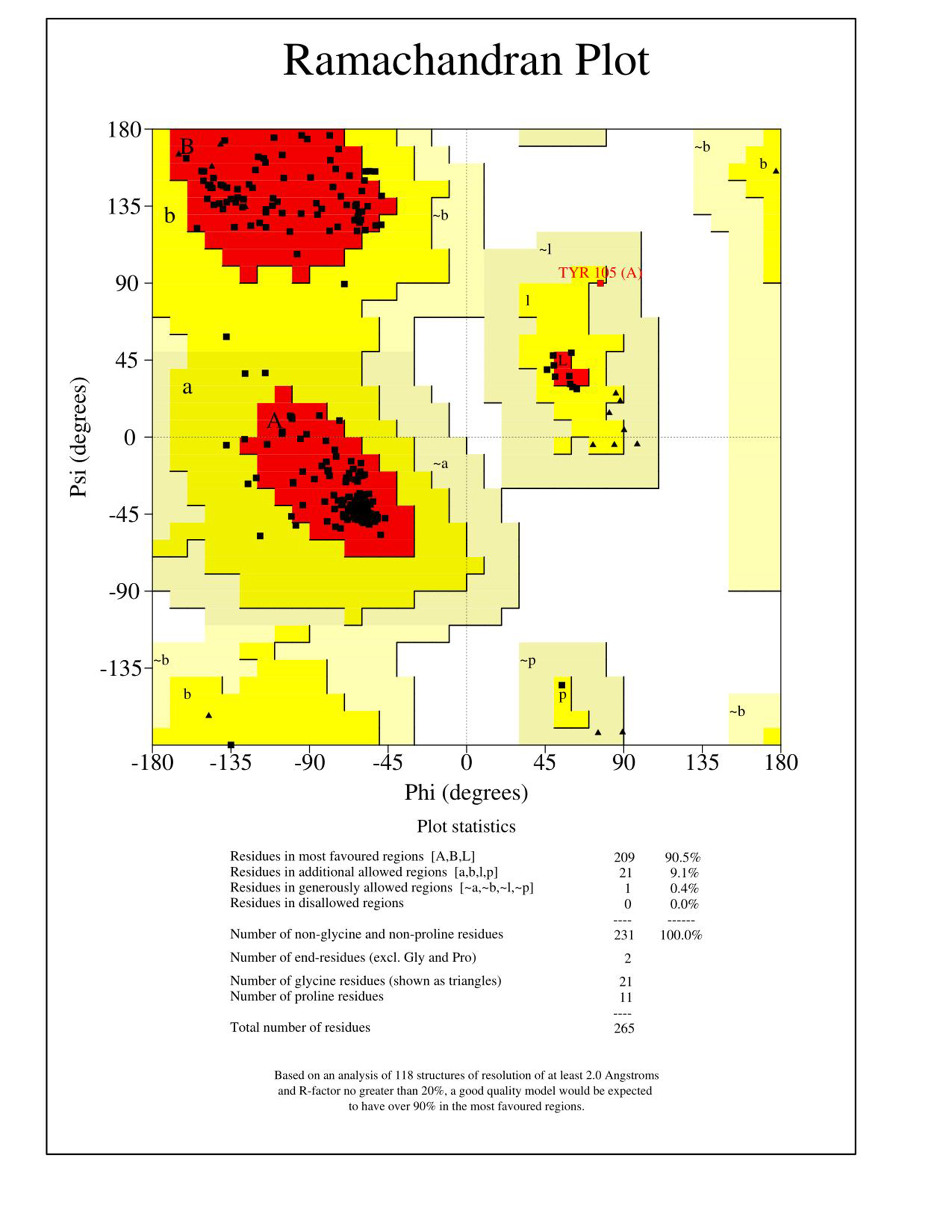

Supplement: Supplementary Figure 5 — RC plot analyses for the modeled SHV-228 protein structure. [file Image_5.JPEG]

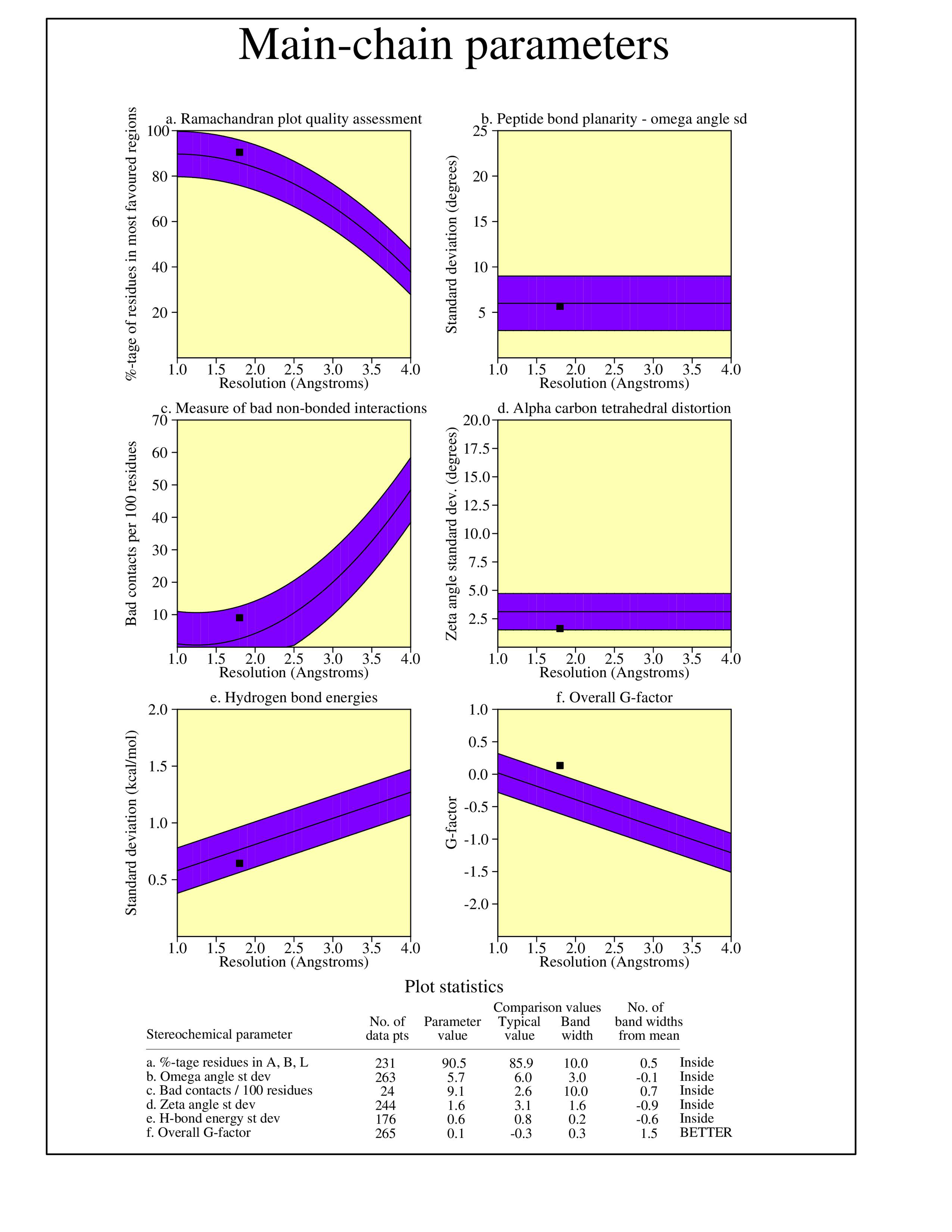

Supplement: Supplementary Figure 6 — Summary of main chain parameters for the modeled SHV-228 protein structure. [file Image_6.JPEG]

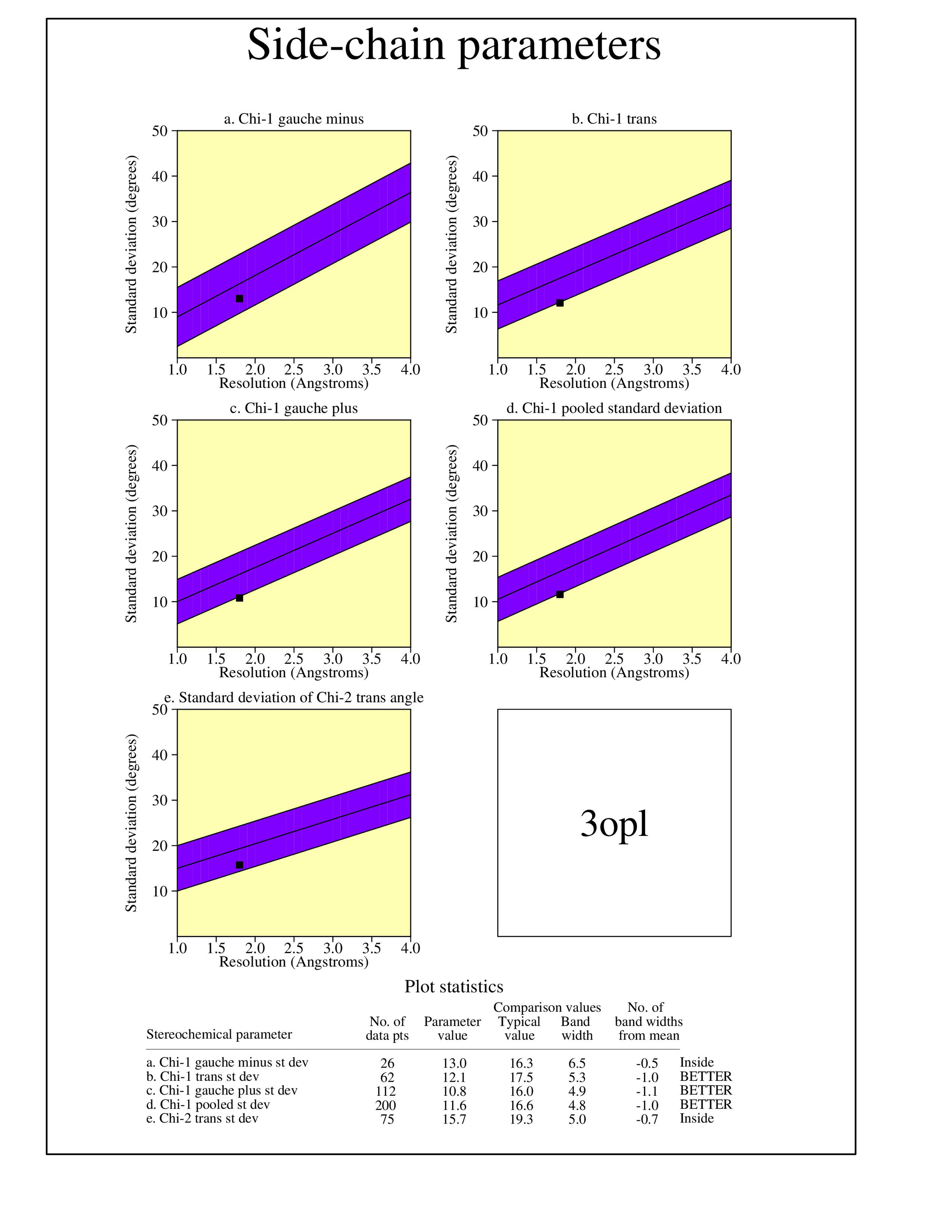

Supplement: Supplementary Figure 7 — Summary of side chain parameters for the modeled SHV-228 protein structure. [file Image_7.JPEG]

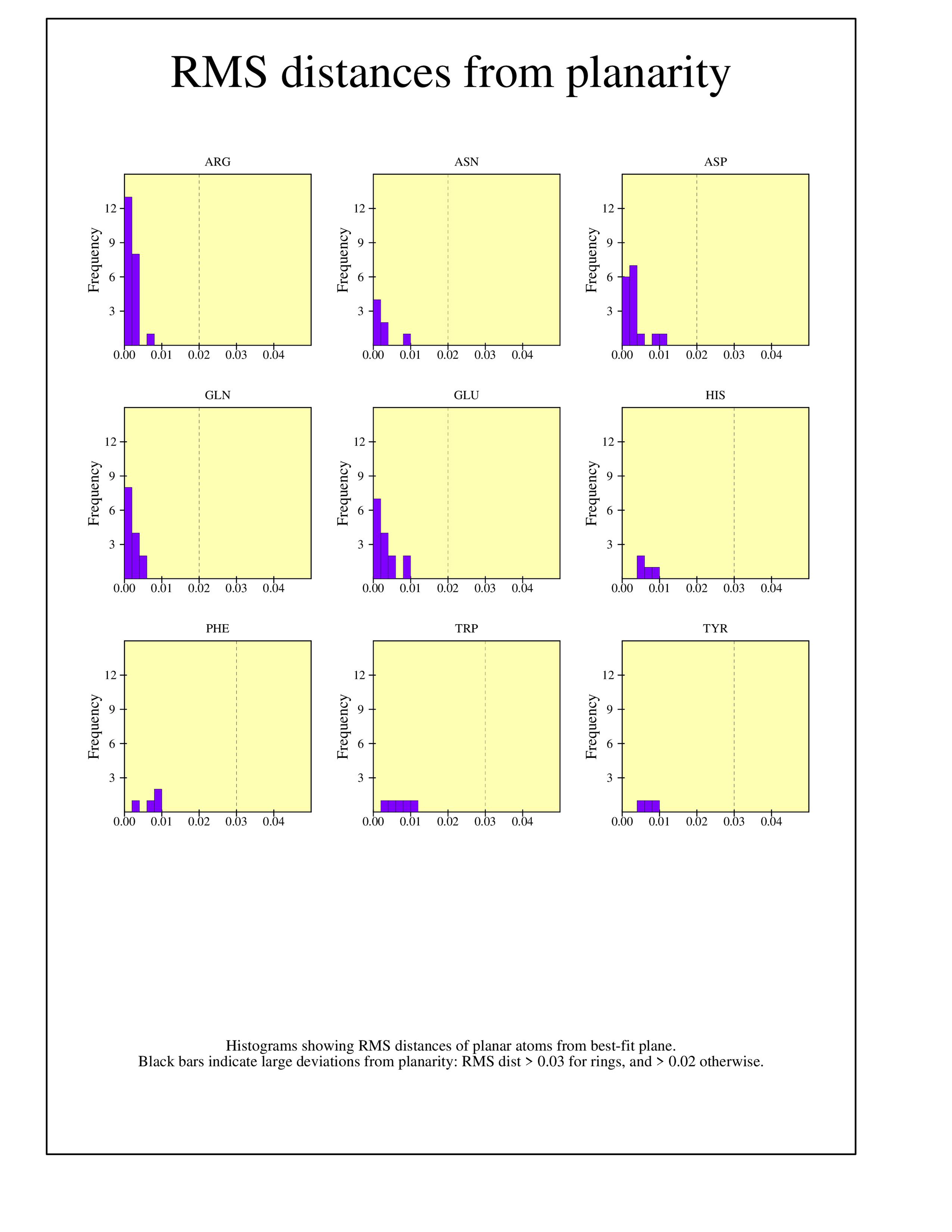

Supplement: Supplementary Figure 8 — RMSD profile for the modeled SHV-228 protein structure. [file Image_8.JPEG]

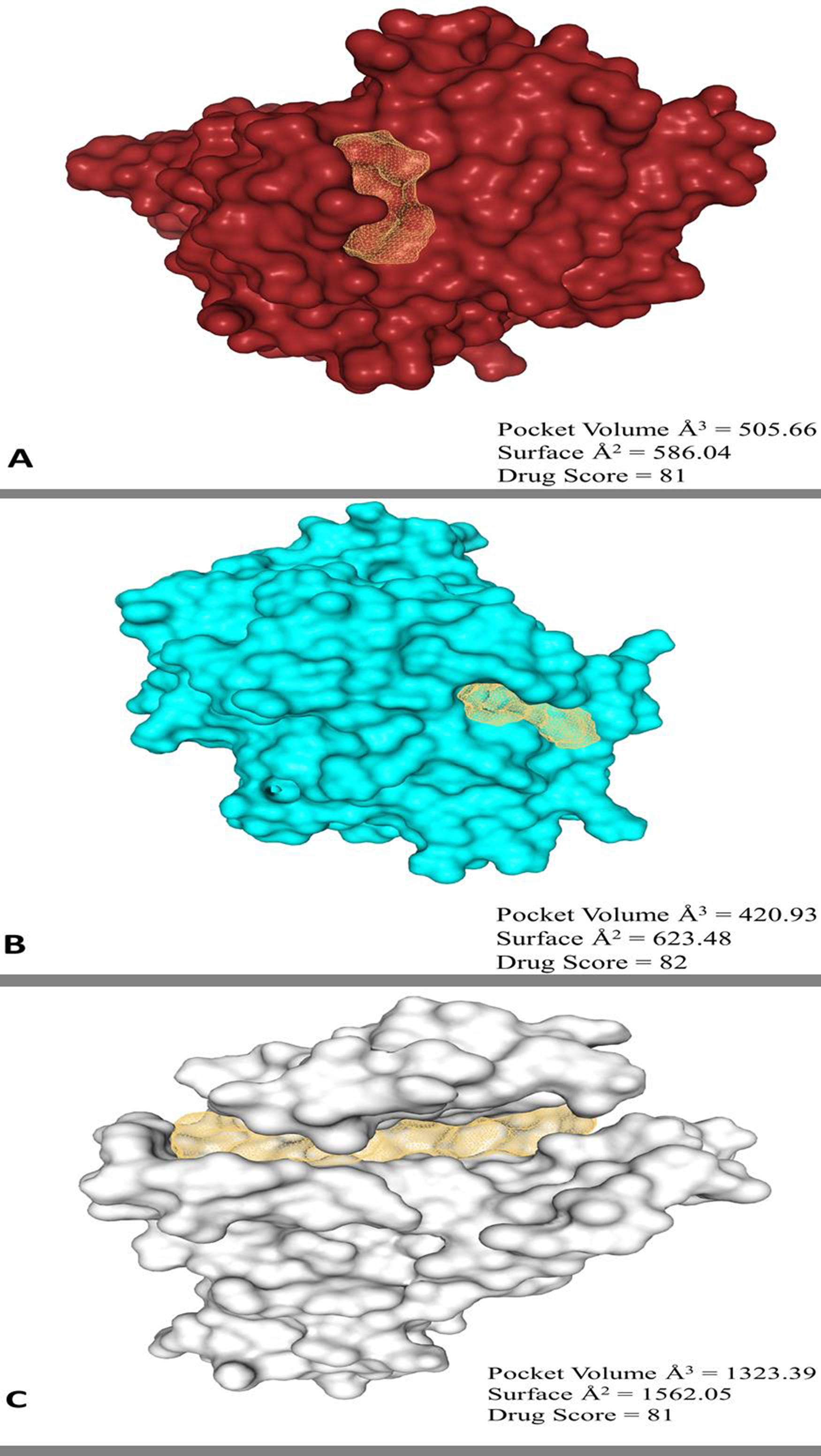

Supplement: Supplementary Figure 9 — Surface view of macromolecules. (A) TEM-1 (in ruby color) showing the opening of the catalytic pocket marked in yellow color. (B) CTX-M-15 (in cyan color) showing the opening of the catalytic pocket marked in yellow color. (C) SHV (in gray color) showing the opening of the catalytic pocket marked in yellow color. [file Image_9.JPEG]
